# Micro-pursuit: A class of fixational eye movements correlating with smooth, predictable, small-scale target trajectories

**DOI:** 10.1167/jov.21.1.9

**Published:** 2021-01-14

**Authors:** Kevin Parisot, Steeve Zozor, Anne Guérin-Dugué, Ronald Phlypo, Alan Chauvin

**Affiliations:** 1 CNRS, Institute of Engineering, GIPSA-lab & LPNC, University of Grenoble Alpes, Grenoble, France; 2 CNRS, Institute of Engineering, GIPSA-lab, University of Grenoble Alpes, Grenoble, France; 3 CNRS, Institute of Engineering, GIPSA-lab, University of Grenoble Alpes, Grenoble, France; 4 CNRS, Institute of Engineering, GIPSA-lab, University of Grenoble Alpes, Grenoble, France; 5 CNRS, LPNC, University of Grenoble Alpes, Grenoble, France

**Keywords:** fixational eye movements, micro-pursuits, micro-saccades, maximally projected correlation, attractor-based model

## Abstract

Humans generate ocular pursuit movements when a moving target is tracked throughout the visual field. In this article, we show that pursuit can be generated and measured at small amplitudes, at the scale of fixational eye movements, and tag these eye movements as *micro-pursuits*. During micro-pursuits, gaze direction correlates with a target's smooth, predictable target trajectory. We measure similarity between gaze and target trajectories using a so-called *maximally projected correlation* and provide results in three experimental data sets. A first observation of micro-pursuit is provided in an implicit pursuit task, where observers were tasked to maintain their gaze fixed on a static cross at the center of screen, while reporting changes in perception of an ambiguous, moving (Necker) cube. We then provide two experimental paradigms and their corresponding data sets: a first replicating micro-pursuits in an explicit pursuit task, where observers had to follow a moving fixation cross (Cross), and a second with an unambiguous square (Square). Individual and group analyses provide evidence that micro-pursuits exist in both the Necker and Cross experiments but not in the Square experiment. The interexperiment analysis results suggest that the manipulation of stimulus target motion, task, and/or the nature of the stimulus may play a role in the generation of micro-pursuits.

## Introduction

Eye movements are typically classified at macroscopic scale as fixation, pursuit, saccade, or reflexive eye movements. But even during fixations, eyes never stay still, and a variety of fixational eye movements have been observed and studied (Martinez-Conde, Macknik, & Hubel, 2004). As an example, micro-saccades have been defined as small-amplitude, ballistic movements, similar to large-scale saccades (Rolfs, 2009). Based on the hypothesis that eye movements are consistent observations in an oculomotor continuum (Otero-Millan, Macknik, Langston, & Martinez-Conde, 2013), and in line with micro-saccades, one can thus expect to observe small-amplitude pursuits within fixations. Here, we will focus on this subclass of slow fixational eye movements, which we term *micro-pursuit eye movements*. We provide evidence of micro-pursuit eye movements at a fixation level, with an adapted metric that reveals their existence. Three different experiments are presented, two where micro-pursuit occurs and one where it does not. In what follows, we will first describe the current classes of macro-scale eye movements, with their functions and metrics, to provide a starting point for the oculomotor continuum hypothesis that we defend.

The main function of eye movements is to orient the gaze toward parts of a visual scene (Yarbus, 1967; Palmer, 1999; Barnes, 2011). To accomplish this goal, the human oculomotor system has the capacity to generate a wide variety of movements that can be categorized based on their spatiotemporal dynamics: amplitude, velocity, and acceleration.

### 

#### 

##### Rapid and ballistic eye movements (saccades)

Classified based on displacement, speed, and acceleration thresholds, for example, displacement above 0.15 degrees (deg), velocity above 30 deg.s^−1^, and acceleration above 9,500 deg.s^−2^, though other detection criteria exist (Nyström & Holmqvist, 2010; Behrens, MacKeben, & Schröder-Preikschat, 2010; Mihali, Opheusden, van, & Ma, 2017). These criteria have become their definition. But absolute threshold criteria have been criticized for their lack of functional, physiological, or formal justifications. For example, the clear dichotomy between fixations and saccades has been loosened (Ko, Poletti, & Rucci, 2010).

##### Slow eye movements (smooth eye pursuits, slow oculomotor control)

Classified based on a simple velocity criterion, for example, smooth pursuit ranges from 20 to 90 or 20 to 100 deg.s^−1^ (Krauzlis, 2004; Komogortsev, & Karpov, 2013; Spering & Montagnini, 2011), though pursuits are considered smooth and precise only at speeds up to 30 deg.s^−1^. If target velocity is too high for the pursuit system, catch-up saccades can compensate for the accumulated position error created by the difference between target and gaze velocities, also known as the retinal slip (De Brouwer, Yuksel, Blohm, Missal, & Lefre, 2002).

##### Eye fixations

Usually defined as any eye movement with an amplitude below 1 deg. They specifically include fixational eye movements that form a generic class of small-amplitude eye movements (ocular drift, tremor, and micro-saccades) sharing dynamic characteristics with regular (macro) eye movements at smaller scale (Otero-Millan, Macknik, Langston, & Martinez-Conde, 2013; Krauzlis, Goffart, & Hafed 2017).

The article is organized as follows: First, slow eye movements are described and associated with their dimension and metrics. Second, small-amplitude, slow eye movements and their dependencies on the visual stimulation, the task, and the experimental paradigm are detailed as well as the metrics used for their detection. Then, we introduce a metric for target-dependent eye movement, maximally projected correlation (MPC), a scale- and translation-invariant metric that measures similarity between the gaze and a target two-dimensional motion during small-amplitude smooth movement. Finally, we propose three experiments and their results: a first experiment (Necker) that allows for the detection of micro-pursuit and two other experiments (Square and Cross) that have been built to replicate the generation of smooth pursuit with different stimuli and tasks.

### Slow eye movements: Different kinds of motion

The functional role of (smooth) pursuit is to maintain a—usually moving—target of interest on the high acuity foveal region of the retina (Spering & Montagnini, 2011). Tracking is believed to be controlled by retinal errors, the difference between gaze and target positions, or retinal slip,^1^ that is, $q_{R}≐q_{G}-q_{S}$, the difference between gaze and target velocities or speed vectors of the gaze and of the target stimulus, that is, ${\dot{q}}_{R}≐{\dot{q}}_{G}-{\dot{q}}_{S}$. According to Orban de Xivry and Lefevre (2007), pursuit relies mostly on reducing retinal slip and is modulated, in a smaller way, by position and acceleration errors.

In order to detect and measure the quality of slow eye movements, metrics have been defined that associate gaze with the target stimulus position. For smooth pursuit, tracking quality is measured through *gain* (see Micro-pursuits section for more details). This measure has shown its effectiveness in experimental protocols where a target appears on screen and participants are tasked to follow its motion. Pursuit is mostly studied for tracking a single point on a uniform background, although other stimuli in motion also lead to pursuit movements, for instance, random-dot kinematograms (Heinen & Watamaniuk, 1998), line figures (Masson & Stone, 2002), illusory perceptual motion (Madelain & Krauzlis, 2003), or after-effect motion (Braun, Pracejus, & Gegenfurtner, 2006). In tasks where a percept is pursued, rather than a stimulus, the measure of gain and the associated models have been questioned (Stone, Beutter, & Lorenceau, 2000).

Among the slow eye movements, we also find reflexive movements such as the vestibulo-ocular reflex (VOR), the oculo-following reflex (OFR), or the opto-kinetic nystagmus (OKN). The VOR is a reflex eye movement that compensates head motion in order to maintain a stable retinal image. Though the VOR expression may be similar to pursuit, it is only generated when the head is free to move. The OFR is a reflexive eye movement in response to a sudden change of a wide-field image (Michalski, Kossut, & Żernicki, 1977; Miles, Kawano, & Optican, 1986; Gellman, Carl, & Miles, 1990; Quaia, Sheliga, FitzGibbon, & Optican, 2012). The reflex is mainly attributed to the tracking of motion in peripheral vision (Ilg, 1997). The OKN is a composite gaze pattern in which an object is followed by smooth pursuit until the object leaves the visual field. At this point, the gaze returns to the object's initial position (fast saccadic response) at the starting position of the pursuit. VOR, OFR, and OKN are eye movements solicited in specific visual stimulation and experimental contexts, which require the manipulation of a large part of the visual field, not a smaller perceptual target, as with pursuit.

To summarize, pursuits have been studied as large-scale eye movements with amplitudes exceeding 1 deg (60 min-arc) in which a target with motion is tracked by the gaze, such that the retinal slip is minimized. The metric used to measure pursuit has been velocity gain.

### Do small-amplitude pursuits exist?

#### Fixational eye movements

We have just described the three principal classes of macroscopic eye movements, where saccades and pursuits are distinguished from fixations based on the amplitudes and velocities involved. However, the fact that during the fixation, the eye never stands still (Ditchburn & Ginsborg, 1953) and continuously produces fixational eye movements further subdivides *fixations* into the following subclasses (Kowler, 2011): *Micro-saccades* are ballistic small amplitude and fast gaze shifts (Rolfs, 2009; Poletti & Rucci, 2016). *Slow drifts* are small-velocity (< 0.5 deg.s^−1^) displacements of the gaze (Nachmias, 1961; Yarbus, 1967), and *tremors (or physiological nystagmus)* are aperiodic high-frequency oscillations of the eye (30–80 Hz and amplitudes of up to 50 s of arc) (Nachmias, 1961; Martinez-Conde, Macknik, & Hubel, 2004). Research has also been conducted on tremor, but due to their small amplitude and high frequency, it is impossible to distinguish them from noise using video-based eye-trackers (Ko, Snodderly, & Poletti, 2016). Therefore, tremors will not be considered in our study. The class of slow drifts, and more particularly small-amplitude pursuits, seems less covered in the literature, which can be explained by the technical difficulties associated with eye-tracker precision, especially video-based ones, at such small scales (Wyatt, 2010; Choe, Blake, & Lee, 2016). As we want to focus on the latter, we will give a detailed review of literature on slow drifts, small-amplitude movements.

#### Micro-saccades

Micro-saccade is a class of fixational eye movements characterized by (i) ballistic properties—like saccades— (ii) small amplitudes, and (iii) a linear relationship between peak velocity and amplitude, also known as a main sequence (Bahill, Clark, & Stark, 1975). The latter stipulates that as micro-saccades have larger amplitudes, their associated (measured) peak velocity increases, and this relationship is linear. In essence, the fast, ballistic nature of micro-saccades allows quickly—typically under 80 ms—repositioning the fovea in the context of visual perception (Rolfs, 2009; Ko, Poletti, & Rucci, 2010; Poletti & Rucci, 2016; Sinn & Engbert, 2016), similar to saccades at larger scales (i.e., not contained within fixational eye movements). Physical properties of the oculomotor system constrain these ballistic movements of the eye to exhibit the linear peak velocity–amplitude relationship.

The main sequence has been very reproducible, and appears in over decades of eye movement research (Rolfs, 2009; Hicheur, Zozor, Campagne, & Chauvin, 2013). Other than providing insight into the oculomotor control system's properties (Bahill, Clark, & Stark, 1975), it also supports the hypothesis of an oculomotor continuum (Rolfs, Kliegl, & Engbert, 2008; Sinn & Engbert, 2016). In Engbert and Kliegl (2003), detection of micro-saccades is based on a lower-velocity threshold computed relatively to the overall velocities in an observation window. As such, the detection threshold is dependent on the contextual oculomotor activity. This is combined with a binocularity criterion to avoid spurious detections. This is also the approach we have followed in this work.

#### Ocular drift: A simple random process or stimulus dependent?

These slow and small movements are the consequence of a slow control system of eye position (Cunitz, 1970) described in literature as a mere drift of the eye (Dodge, 1907), OFR (Chen & Hafed, 2013), or—more recently—as small-amplitude pursuits (Skinner, Buonocore, & Hafed, 2018).

In early studies of fixational eye movements, when subjects had to fixate a static dot, eyes drifted slowly with an upper velocity limit at 0.5 deg.s^−1^ and mean velocity of 5 min-arc.s^−1^ (Yarbus, 1967). Their trajectories were considered random and involuntary processes since they showed dynamics similar to Brownian random walks (Ratliff & Riggs, 1950; Engbert & Kliegl, 2004) as well as independence between the two eyes (Cornsweet, 1956). However, Ditchburn and Ginsborg's (1953) work provided evidence that direction of eye movement is not completely random during drift; it is idiosyncratic. Nachmias (1961) replicated this finding in an experiment where a fixation target was switched on and off during 3-s cycles. He found that each of the two subjects have preferred drifting direction, but this preferred direction can be modified by changing the visual environment. The author interpreted the idiosyncratic direction preference as specific to muscular response and reasserted that nonrandom ocular drifts occur in fixations while providing evidence that drift direction can be modulated by the visual environment. More recently, a variety of experiments have shown that drift can take properties and characteristics close to other known oculomotor phenomena (Poletti, Listorti, & Rucci, 2010; Chen & Hafed, 2013; Skinner, Buonocore, & Hafed, 2018; Watanabe, Okada, Hamasaki, Funamoto, Kobayashi, & MacAskill, 2019).

As mentioned, drift can be viewed as part of a slow control system, enabling gaze to capture a target, whether static or dynamic. Here, we will discuss two studies that show evidence of slow eye movements correlating with the target stimulus and as such related to our proposition of adding a subclass to the fixational eye movements: that of micro-pursuits.

Chen and Hafed (2013) studied the impact of micro-saccades on visual perception and investigated the relationship between micro-saccades and drift. Their experiment contained two major tasks. The first task required two monkeys to stare at a fixation dot where a change in luminance of the dot or a peripheral white flash was introduced to induce a higher probability of micro-saccade generation. Drift velocity was analyzed before and after the micro-saccades using either direct velocity measurements or spatial dispersion (by spatial binning and box counts). Both measures showed an increase in drift velocity post-micro-saccadic movements with respect to pre-micro-saccadic movements or baseline movements. They also showed that eye drift mainly occurs in the direction opposite to the micro-saccade, which is interpreted as corrective slow control of the gaze position. The second task consisted of a sinusoidal grating that started moving at predefined delays after the onset of a micro-saccade (or after 500 ms if no micro-saccade was detected). The authors analyzed the speed and direction of early drift of the eye, namely, the OFR, according to the direction of the grating and the time of grating onset based on micro-saccade detection. Indeed, they reported that (i) the drift directions were in the opposite directions of the micro-saccades and (ii) the eye velocity was reduced when the grating's motion was initiated during micro-saccade and was enhanced when the motion was initiated after micro-saccade. Since ORF is an indicator of “the sensitivity of early motion processing to retinal-image slip after a micro-saccade,” the OFR and thus motion perception, are suppressed during the saccade and enhanced after. Their overall findings suggest that there is a single slow gaze control system that controls both fixation and eye movement position in the presence of a fixed target or a slow-moving background linked to the motion perception system. Conclusions suggesting a subtle coupling between micro-saccades and drifts are also reinforced by previous reports (Engbert & Mergenthaler, 2006).

Part of this idea had already put forward by Murphy and colleagues (Murphy, Kowler, & Steinman, 1975). In their experiment, they asked participants to maintain their gaze on a present or absent fixation dot while a grating in the background moved horizontally at velocity ranging from 0.08 to 8 deg.s^−1^. In a second condition, the participants had to follow the grating. Eye movement velocities were analyzed for trials without saccades. The study shows that when participants have to stare at the fixation dot, (i) they have an ability to keep gaze fixed when the fixation dot was present, and (ii) an OFR—a smooth displacement of the eye in the direction of the grating's movement but with smaller velocities—is detected when the fixation dot was absent. In contrast, when the task was to follow the grating, participants showed clear smooth, slow movement in the direction of motion with velocity as low as 0.08 deg.s^−1^.

Both these studies confirm the existence of a slow movement within a fixation that tracks a slow-velocity target or counteracts the displacement of a micro-saccade. These slow movements of pursuit or fixation stabilization are thought to be under a same slow control system, although the tracking mechanism seems not to be triggered when the movement is initiated during a micro-saccade.

#### Ocular drift and slow motor control

Drift has been linked to slow control of the eyes during fixation in the context of investigating links between visual stimulation and drift motion.

In a series of experiments, Kowler and Steinman (1979) have investigated how expectation, over a stimulus and task, can induce anticipatory smooth and slow eye movements. The authors implemented a task in which participants had to track a dot moving by steps (with three frequencies: 0.25, 0.375, or 0.5 Hz) along a horizontal segment of 3.3 deg of amplitude. They showed that eye movements’ direction and latency depend on predictability of target displacement. Furthermore, they showed this effect to remain even when the level of predictability was manipulated and when a distracting secondary task was imposed (Kowler & Steinman, 1981). In fact, they provided evidence that anticipatory eye movements—which they also named involuntary drifts in the direction of future target motion—depended on the history of prior target motions (Kowler, Martins, & Pavel, 1984). To understand whether the slow control of ocular drift is driven by position or velocity signals, they carried out an experiment in which they manipulated drift by changing the configuration of reference points, thus varying the difficulty of fixation of a central point (Epelboim & Kowler, 1993). Their analyses used gaze position data and bivariate contour ellipse area (BCEA) computation for quantification of gaze dispersion. As such, they provided evidence that the oculomotor system does not rely on visual position signals, but rather on retinal image slip, in order to implement slow motor control. This creates a parallel with the known models for smooth eye pursuit described above.

In addition, in a recent article, Watanabe and colleagues (2019) reported a study that links ocular drift, micro-saccades, and pupil area on voluntary eye movements' preparation. They observed anticipatory drifts prior to stimulus appearance, and they argue that these anticipatory eye movements may reflect volitional action preparation. Interestingly, the authors provide a replication of previous results on anticipatory drift with a video-based eye-tracker while applying correction to their gaze signals for pupil deformation.

Overall, these studies show that slow eye movements are present during fixation. These movements can control for a fixation position, can track large targets, and depend on expectation. Authors have postulated that all these behaviors are under control of a unique system.

#### Small-amplitude pursuits

As mentioned, higher, smooth pursuits are large-scale eye movements with amplitudes exceeding 1 deg (60 min-arc). A small set of studies found eye movements within a fixation that share characteristics with smooth pursuits, except for their amplitude. Though there are references to smooth pursuits of small amplitude as far back as (Yarbus 1967), most studies in the literature have reported the phenomenon in an indirect manner.

In a study on drift in the absence of visual stimulation or with afterimages, horizontal smooth drifts were reported (Heywood & Churcher, 1971). Although their description corresponds to pursuit dynamics, they did not define the observed movements as such. The authors published a follow-up article showing that, depending on the eccentricity of the afterimage, oculomotor dynamics are more or less smooth and show low velocities, and hence they could be interpreted as pursuits (Heywood & Churcher, 1972). Further, while attempting to study oculomotor control capacities when presenting a moving grating background with a fixation point, Murphy and colleagues (Murphy, Kowler, & Steinman, 1975) reported eye movements that correspond to small-amplitude pursuits. When investigating the lack of compensation of the VOR when the head was free, Martins, Kowler, and Palmer (1985) studied whether a smooth pursuit system might interact with the VOR. Their data provided a qualitative description that small-amplitude pursuits are related to the velocity of target motion. The following finding was reported: Foremost, the effectiveness of smooth pursuits varied with target velocities. At the lowest average velocities of a tracked point^2^ (0.0025—0.125 deg.s^−1^), smooth pursuit was the most effective, that is, retinal image speed during smooth pursuit was about the same as retinal image speed during low-target velocities. At higher-target velocities (0.25–1 deg.s^−1^), smooth pursuit was less effective for retinal image stabilization, and at the highest velocities (1.5–2.5 deg.s^−1^), smooth pursuit was totally ineffective.

More recently, small-amplitude pursuits have been reported again, in very different contexts. In a study of eye drift and its relationship to retinal image motion—investigating whether the latter drives the former through retinal or extra-retinal information—Poletti and colleagues (Poletti, Listorti, & Rucci, 2010) declared the following observation: “small pursuit-like eye movement with amplitudes comparable to those of fixational drifts are under precise control of the oculomotor system.” Finally, a precise characterization of rhesus macaque oculomotor control for rectilinear sinusoidal motion of a target with amplitudes inferior to 0.5 deg and velocities below 2.5 deg.s^−1^ was recently reported (Skinner, Buonocore, & Hafed, 2018). The amplitude and frequency of the sinusoidal motion was modulated and gaze signals were analyzed using gain and compared to filter responses; filters are, here, used as models to show how the oculomotor system could display different behaviors based on input frequencies—on gaze position and velocity. Furthermore, they showed that the gaze signals had eye velocity spectrum with peaks at target frequency and that pursuit gain was highest at 1 deg.s^−1^.

Overall, *pursuits* have been observed for a range of velocities (0.05–2 deg.s^−1^) and amplitudes (1.9–30 min-of-arc), which qualifies them as fixational eye movements. Given the classification in the fixational eye movements research field—in which only micro-saccades, drifts, and tremors are considered—these observations raise questions on the nature and potential definition of micro-pursuits or fixational pursuits.

This article focuses on the presentation of micro-pursuits in three contexts: (i) presentation of metrics that fit the theoretical requirements to detect micro-pursuit and (ii) detection of the oculomotor phenomenon in (a) a dual-task experiment (Necker) in which its elicitation was not explicitly made to participants and (b) an explicit tracking experiment (Cross) and an implicit distractor setup (Square). Our hypothesis was that if the perceptual system has to detect a change in a moving stimulus with a predictable trajectory, the oculomotor system is likely to follow the target even if the participant is instructed not to do so (fixation task). But since the fixation task inhibits large deviations, only small-amplitude pursuit eye movements are generated. Furthermore, a computational model of pursuit eye movements based on gravitational energy fields is presented in the Appendix C that accounts for the two contrasting objectives (fixation vs. pursuit). In our data analyses, we made use of a measure of inertia for gaze dispersion and MPC for similarity, since they are simple methods that showcase clear advantages in our context. The latter also offers a metric that can be physically interpreted as it is able to capture similarity between two trajectories of different scales and spatial offsets.

## Micro-pursuits

The study of micro-pursuit should aim to find consistent characteristics—like the main sequence for the micro-saccade—that can be measured through an adequate metric. Micro-pursuit being a slow eye movement, exhibiting strong similarity with the target (stimulus) trajectory, we will consider a fixation to be of the class micro-pursuit whenever the above criteria are met. In addition, if the oculomotor continuum holds true, these slow movements potentially alternate with small ballistic movements, called catch-up saccades, as is the case at macroscopic scale. It is clear that a thorough study of micro-pursuits thus needs a full characterization of fixational eye movements (especially micro-saccades) as well as the evaluation of a similarity measure between gaze and target.

### Quantifying pursuit movements (metrics)

To propose a definition of micro-pursuit movements, existing metrics for ocular movements will be discussed, since they will orient our choices for proposing metrics and hence our working definition.

Classical smooth pursuit is measured by retinal slip gain ($\text{gain}=∥\dot{q_{G}}∥/∥\dot{q_{S}}∥$ with $\dot{q_{G}}$ the gaze velocity and $\dot{q_{S}}$ the stimulus velocity), which is consistent with its closed-loop modeling (Barnes, 2011). Position gain is also used, although to a lesser extent, for instance when dealing with catch-up saccades (Orban de Xivry & Lefevre, 2007). For the various drift phenomena described in the previous section, a variety of metrics have been used to study fixational eye movement dynamics (e.g., gaze position, velocity, acceleration, gain, and BCEA). For instance, gain measurement was used for analysis in the case of the small-amplitude pursuits of monkeys on univariate sinusoidal motion (Skinner, Buonocore, & Hafed, 2018). But the authors went further and provided a spectral analysis using Fourier transform on eye signals to identify the fundamental frequency and harmonics with the expected target frequencies. However, gain is a univariate metric that does not extend to multivariate problems. Thus, it can be used adequately only for pursuit of a target moving on a line, rather than a plane, like the visual field. Fourier analysis shares the same issue as it looks for a frequency in a univariate movement, typically horizontal.

In studies of ocular drift (Epelboim & Kowler, 1993), BCEA^3^ was used to quantify the spatial variance—inertia, or spread—of the gaze. The authors obtained orientation preferences through the inferred relative anisotropy of the ellipse. Though this metric is clearly conceived for bivariate signals, it does not provide spatiotemporal correlation between gaze and a target signal in the way gain does. Meanwhile, the box-count method used in more recent studies permits computing dispersion of the gaze data over time, though it may suffer, like gain, from measurement noise, especially with the video-based eye–tracker (Engbert & Mergenthaler, 2006; Chen & Hafed, 2013). To summarize, (i) some metrics, for example, BCEA, box count, and inertia, can be used as quantifiers for the spread of a bivariate gaze signal during an epoch, and these metrics are useful descriptors for drift and slow movements, and (ii) other metrics, for example, gain, Fourier analysis, and correlation, can be used to quantify similarity between two bivariate signals, to quantify the quality of a pursuit between gaze and a stimulus in motion. Each metric presents a trade-off that should be considered based on a theoretical definition and prediction.

### Micro-pursuits: A working definition

Given the reported observations of small-amplitude pursuits, the following constraints need to be considered to define a *micro-pursuit*.

#### 

##### Amplitude

As indicated by the prefix of its name, and as an analogy to micro-saccades, the micro-pursuit must be of small amplitude, within the range of fixational eye movements, typically below 1 deg.

##### Velocity

Micro-pursuit should consist of slow eye movements, similarly to drift, or smooth pursuit but at a smaller scale, with velocities below 2 deg.s^−1^.

##### Tracking

Micro-pursuits occurs when a percept with motion across the observer's visual field is tracked. But, as pursuit involves matching the motion of a target to that of an observer in real time, micro-pursuit measurement of tracking should reflect the spatiotemporal interaction between the dynamics of two bivariate signals. Hence, *similarity* between gaze dynamics should be evaluated. Because the eye movement amplitude is within the fovea's size, deformation may occur in the tracking of predictable bivariate signals. Therefore, any similarity metric should exhibit both scale and translation invariances—spatial offset invariance may also be beneficial for measures from eye-trackers with lower precision and accuracy.

##### Duration

The phenomenon of tracking a moving target requires by definition that it is done over a sufficiently long epoch. Thus, micro-pursuit should not occur over brief epochs such as saccades and micro-saccades.

##### Binocularity

Conjugated movements on both the guiding and the complementary eye can be expected, being a strong indicator of oculomotor planning.

We propose that gaze signal epochs satisfying the above description be considered *micro-pursuits*. As this is a proposed working definition, micro-pursuits may correspond to entire eye fixation periods, making it possible for micro-pursuit to be punctuated by other fixational eye movements. Once its properties are defined more precisely than above and detection algorithms can be developed, it will be possible to discriminate micro-pursuits from other fixational eye movements, like micro-saccades.

### Descriptive statistics for the classification of micro-pursuits

Choosing an adequate metric for analysis was key, given the constraints presented in the previous section and our experimental setup. Two metrics, *inertia* and MPC, are used in this work; they provide complementary information about the data. The first is a measure of the spatial dispersion of the gaze within a fixation to investigate the marginal dynamics of the gaze during fixational eye movements. The second metric gives a quantification of similarity—and hence interaction—between the gaze and a target. Compared to works in the literature with similar observations (Martins, Kowler, & Palmer, 1985; Skinner, Buonocore, & Hafed, 2018), an essential aspect was to have a metric that could reflect similarity with noise robustness, as well as scale and translation invariance. Moreover, this was needed in the context of movements in the plane, rather than rectilinear ones for which uni-variate measures are sufficient. A benefit from such considerations is to propose a generalized metric for micro-pursuit that could be applied to track perceived motion in the two-dimensional visual field projected on the retina. MPC offers a method to quantify spatiotemporal similarity between two bivariate signals. Furthermore, inertia and MPC can both be applied on the gaze signals in fixation epochs detected by the video-based eye-tracker algorithm. Their mathematical relationship is detailed more in depth in Appendix B.

### Measuring gaze dispersion with inertia

The dispersion of gaze within a fixation was computed using a measure of inertia, a metric used to quantify the spread of a cloud of data points with respect to a fixed point, usually its empirical mean. Here, we used a similar but generalized formula based on the mean quadratic distance from an arbitrary reference point. As such, in the case of stimulus motion, we can compute inertia with respect to the stimulus’ center of gravity. Let ${\bar{q}}_{U}≐\frac{1}{N}\sum_{i=1}^{N}q_{U}^{i}$ be the empirical mean of a signal whose samples (i=1,...,N) are given by $q_{U}^{i}={\left[x_{U}^{i},y_{U}^{i}\right]}^{⊤}$. We will use U=G for the observed gaze and U=S for the coordinates of the stimulus (center of gravity). Gaze inertia I was computed over the stimulus trajectories over a trial as follows:

(1) $$\begin{array}{ccc} I & = & \frac{1}{N}\sum_{i=1}^{N}{\left(q_{G}^{i}-q_{O}^{i}\right)}^{⊤}\left(q_{G}^{i}-q_{O}^{i}\right)\\ & = & \frac{1}{N}\sum_{i=1}^{N}{∥q_{G}^{i}-q_{O}^{i}∥}^{2} \end{array}$$

where N represents the total number of frames in the trial, $q_{G}={\left[x_{G},y_{G}\right]}^{⊤}$ the measured monocular bivariate gaze signal coordinates, and $q_{O}={\left[x_{O},y_{O}\right]}^{⊤}$ the origin reference point coordinates in the screen plane—however, one can compute inertia with respect to other points in space, for example, stimulus center of gravity or the fixation's mean gaze position. Inertia quantifies gaze displacement as does BCEA (Epelboim & Kowler, 1993) and box-count measures (Engbert & Mergenthaler, 2006). Its key advantage over the former two is that inertia is a more intuitive measure of spatial displacement over a fixation period. The box-count metric is simple and provides similar insight in gaze dispersion over an epoch; it is dependent on the size of the box in space and time used for analysis. Hence, it corresponds to a down-sampling measurement of inertia over a fixed time window. Finally, inertia provides the advantage of being a metric relative to a chosen origin or reference point—box count being independent of the origin—and thus it can be used to look at spatial displacement in the following three contexts: (1) absolute inertia ($I_{\text{screen}}$) is obtained by choosing the center of screen as a reference (absolute, like box count; $q_{O}={\left[0,0\right]}^{⊤}$), (2) relative retinal image instability ($I_{\text{stimulus}}$) by choosing the stimulus’ center of gravity (for pursuit; $q_{O}=q_{S}={\left[x_{S},y_{S}\right]}^{⊤}$), and (3) general relative fixational eye movement instability ($I_{\text{fixation}}$) by referring to the fixation center of gravity (obtained by choosing $q_{O}={\bar{q}}_{G}={\left[{\bar{x}}_{G},{\bar{y}}_{G}\right]}^{⊤}$ with ${\bar{q}}_{G}$, the empirical mean of the gaze for an N samples fixation epoch).

### Measuring gaze-stimulus similarity with MPC

Though humans can intuitively express a qualitative judgment of similarity between two trajectories, obtaining a quantified and objective value for any two bivariate signals is not as trivial as one might suppose. Gain, of gaze velocity over stimulus velocity, has been used as a metric in pursuit data analysis (Skinner, Buonocore, & Hafed, 2018), though the stimulus moved in a univariate context: either horizontal or vertical. In bivariate signals, however, a gain will be obtained for each dimension of the signal, and hence some form of projection to obtain a scalar metric is required. Although similarities between the stimulus and gaze trajectories can be quantified with a diversity of metrics, we will here focus on a measure based on multivariate statistical theory (Anderson, 2003; Muirhead, 2009), quantifying the interaction between the stimulus ($q_{S}$) and gaze ($q_{G}$), in order to infer the similarity of their trajectories during fixations. We choose to determine the direction of the plane for which correlation between gaze and target within a fixation is maximized and report the such obtained correlation value, which we call MPC. Our metric hence inherits the ease of interpretability from (Pearson) correlation values and has low computational costs (just as gain). In addition, for unidirectional motion (see, e.g., Skinner, Buonocore, & Hafed, 2018), this exactly corresponds to Pearson's correlation coefficient between the two time series.

Let $\Sigma_{SG}≐\frac{1}{N}\sum_{i=1}^{N}q_{S}^{i}{q_{G}^{i}}^{⊤}-{\bar{q}}_{S}{\bar{q}}_{G}^{⊤}$ be the empirical (variance-)covariance matrix between stimulus (*S*) and gaze (*G*). We then write $\rho^{*}$ as the maximal absolute empirical correlation that can be obtained under simultaneous projections onto a one-dimensional space, that is,

(2) $$\begin{array}{ccc} \rho^{*} & \;≐ & \max_{w}\;\rho \left(w\right)\;\text{where}\\ \rho (w) & \;≐ & \frac{w^{⊤}\Sigma_{SG}w}{\sqrt{w^{⊤}\Sigma_{SS}w}\sqrt{w^{⊤}\Sigma_{GG}w}}\; \end{array}$$

and w represents the coordinates of the vector onto which both the gaze and the stimulus signal are projected. This method projects the data in a new space and provides a quantity bounded between –1 and 1, where 1 shows perfect correlation and –1 perfect anticorrelation. By construction, MPC is invariant with respect to scale and to a translation of either or both of the signals.

To summarize this section, in this work, inertia with respect to screen ($I_{\text{screen}}$) was used as a measure of gaze displacement. Inertia with respect to stimulus ($I_{\text{stimulus}}$) was used as a measure of retinal image displacement. Inertia with respect to fixation ($I_{\text{fixation}}$) was used as a measure of fixational eye movement displacement. And finally, MPC ($\rho^{*}$) was used as a measure of similarity between gaze and stimulus trajectory during a fixation.

## Main experiment: Necker cube

Micro-pursuits were observed and systematically detected at first in an experiment in which a moving ambiguous Necker cube stimulus was presented and participants had to report their perceived orientation. They were instructed to keep their gaze fixed on a static fixation cross at the center of the screen and report which side of the cube was perceived at the front: either lower-left or upper-right square. The main objective of the experiment was to manipulate the rate of reversal by imposing different motion to the cube. In this article, we focus solely on the oculomotor analysis of this data set, because the manipulation failed to induce any change in the reversal rate between the percept and any observable percept modulation.

### Method

#### Apparatus

The display used was a 40-cm x 30-cm (20 in.) VisionMaster Pro 513 screen of resolution 1,024 by 768 pixels and a 75-Hz refresh rate, located 57 cm from the participants, with mean gray luminance at 68 cd.m^−2^. Eye movements were recorded using the Eyelink 1000 (SR Research, Ottawa, Ontario, Canada). Both eyes were tracked with a 1,000-Hz sampling rate. The head was stabilized using a chinrest. A 9-point calibration routine was carried out at the beginning of each task and was repeated at the beginning of each block (every 15 trials) or when drift correction, performed every 5 trials, reported a mean error superior to 0.5 deg.

#### Stimulus and motion conditions

**Figure 1.**
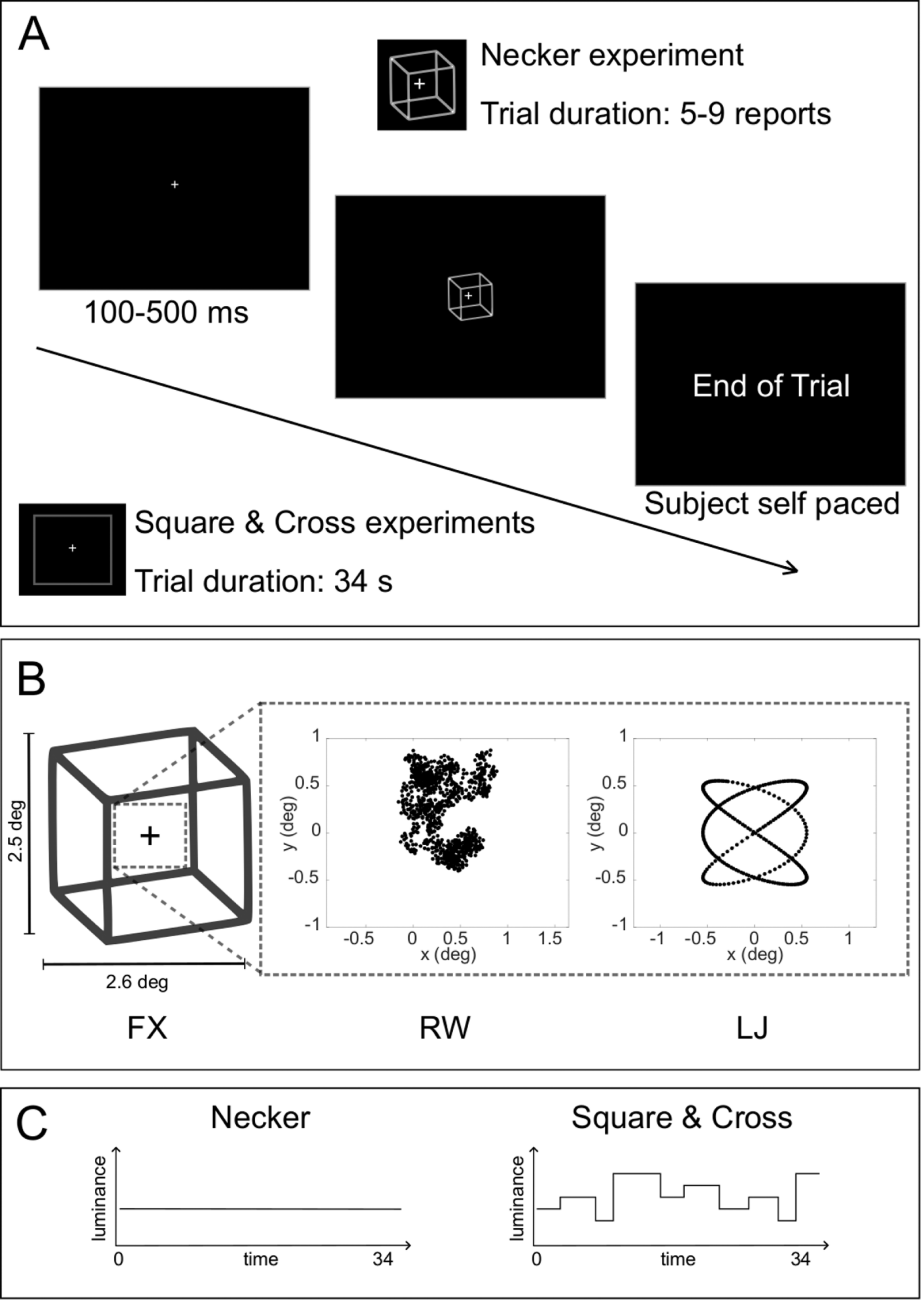
Experimental protocols. (A) A timeline of a trial for all three experiments (Necker, Square, Cross). For the Necker experiment, a Necker cube was displayed and the trial finished if the participant had reported a randomly picked number of perceptual reversals. For the Square and Cross experiments, a plain square was displayed and trial lasted approximately 34 s. A fixation cross was shown during a randomly chosen interval between 100 and 500 ms. (B) The three different stimulus motion conditions: (1) FX, for the control no-motion condition; (2) RW, for the unpredictable random walk condition; and (3) LJ, for the predictable motion based on Lissajous trajectories. (C) Representations of the stimuli's luminance. For the Square and Cross experiments, luminance changed randomly between five levels in order to provide the participants with a perceptual report task, while the Necker cube always kept a constant luminance.

We imposed three types of motion to an ambiguous Necker cube of 2.6 x 2.5 deg (Figure 1A): (1) **FX**, the control condition with no motion; (2) **RW**, an unpredictable motion condition with a random walk; and (3) **LJ**, the predictable motion condition where the cube moved along Lissajous trajectories (see Figure 1B). *Random walk* trajectories were implemented by choosing at each time step an amplitude chosen from an exponential-Gaussian distribution and an orientation from a uniform distribution on (-π,π). The exponential-Gaussian distribution was built from the sum of two independent variables: ϱ=G+E, where G∼N(μ=1.1;σ=0.2) is the Gaussian component, and E∼E(λ=0.1) is the exponential one—units are in pixels (pix) and the ∼ symbol stands for “distributed according to.” A radial limit of 10 pix (0.329 deg) with respect to the center of the screen was implemented so that a step that would exceed the limit would have its orientation reversed such that the step would bounce back toward the center. *Lissajous* trajectories in the LJ condition were defined by x(t)=Asin(cθt) and y(t)=Bsin(dθt+ϕ) with, in our setup, A=B=14 pix (0.5497 deg) and ϕ=0 rad. The Lissajous ratio between signal frequencies was randomly (uniformly) chosen across trials so that (c,d)∈(2,3),(3,2),(-2,3),(-3,2) and $\theta =2\pi \frac{\left(30/2.21\right)}{415}=0.2$ Hz. The parameters’ values were chosen empirically through ad hoc tests.

Stimulus spatial displacement due to movement was controlled across motion conditions. Indeed, their inertia with respect to screen distribution was similar, with RW and LJ generating displacement of the same order of magnitude on average over trials (${\bar{I}}_{\text{screen}}^{RW}=0.2995\pm 0.1988$, ${\bar{I}}_{\text{screen}}^{LJ}=0.2747\pm 0.1372$).

#### Tasks and participants

Twenty-three adults with normal or corrected-to-normal vision (self-assessed) participated in the experiment (15 females and 8 males; age range = 20–71 years, μ=28.35±10.93 years), whose tasks were twofold:

• fixate a fixation cross at the center of the screen for a random interval between 100 and 500 ms (uniform distribution);
• report percept reversals of an ambiguous Necker cube by pressing the arrows of a keyboard when perceptual changes occurred.

The experiment followed a continuous viewing paradigm in which trials had variable (random) durations (μ=34.00±13.26 s; see Figure 1A) and ended based on which of the following conditions happened first:

1. number completion of a trial-based randomly (uniformly) set integer number ($n_{\text{rev}}∼U\left(5,9\right)$) of perceptual reversals on the ambiguous stimulus (see Figure 1A);
2. time-out maximal percept duration of 20 s.

The experiment was programmed using the *PsychToolBox* in MATLAB (Brainard, 1997). All participants gave their informed written consent before participating in the study, which was carried out in accordance with the Code of Ethics of the World Medical Association (Declaration of Helsinki) for experiments involving humans and as approved by the ethics committee of University Grenoble Alpes.

#### Data analysis

##### Data preprocessing

In our data analysis, only fixations of sufficient duration (>80 ms) were considered. The duration threshold was set based on (1) the lack of significant fixations of interest in shorter time windows and (2) the necessity for the MPC metric to have a sufficient number of samples (see Appendix B). Guiding eye gaze signals were first passed through a corrective process to adjust for pupil area deformation, as described in Choe, Blake, and Lee (2016). As the gaze and stimulus signals were systematically compared and computed together, we then applied a Butterworth filter (second-order low-pass filter with a cutoff frequency of $f_{c}=35$ Hz) to smooth the gaze data and down-sampled the gaze signal at the same frequency as the refresh rate of the stimulus (75 Hz). Thus, all analyses were done with data down-sampled from 1,000 Hz to 75 Hz. Fixations generating inertia with respect to screen values beyond two standard deviations from the mean or NaN (due to missing samples) were considered samples with faulty or jittery gaze recording and were removed from analyses. Data for Micro-pursuit analysis and statistical tests only consider fixations without micro-saccades, where the latter are detected by an algorithm proposed by Engbert and Kliegl (2003) based on the binocularity criterion. The algorithm uses relative thresholds based on median absolute deviation of the eye velocity, over a fixation. Data for Micro-saccade analysis and the Focus on MPC results were analyzed, including fixations containing micro-saccades. Outliers were defined as data points^4^ beyond two standard deviations from the mean and were systematically removed from analyses. The results presented do not show these outliers, for better readability, but we also conducted the analyses with the outlier and found the same effects for all tests and experiences.

**Figure 2.**
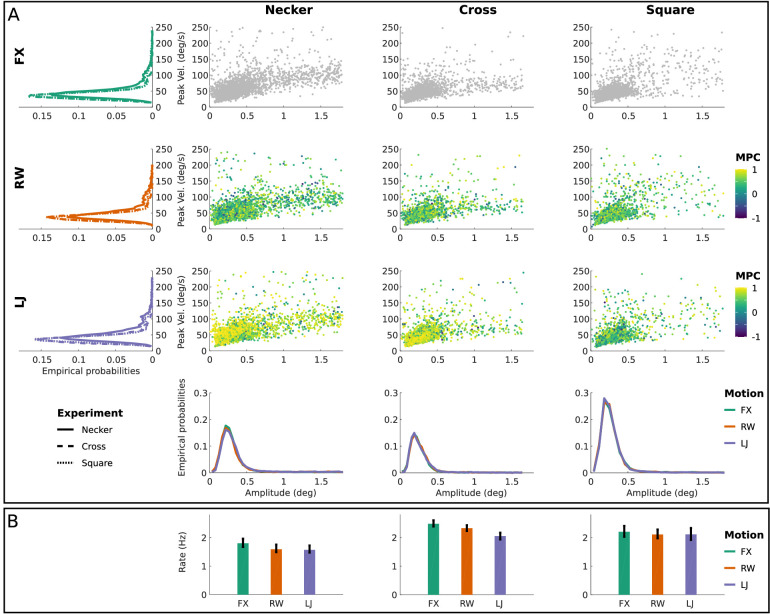
Micro-saccade analysis. (A) The main sequences when plotting micro-saccades’ amplitudes versus peak velocities for all three experiments (Necker, Cross, and Square) and conditions (FX, RW, LJ). The color encodes the micro-saccade's fixation similarity score (using MPC) in the LJ and RW conditions. *Left side*, marginal distributions of peak velocity depending on the experiment and condition are given, while *below*, marginal distributions for amplitudes are shown. (B) Mean micro-saccade rates over experiments and conditions with, in black, 95% confidence intervals computed using bootstrap (n=200 iterations).

##### Statistical methods

Statistical tests were conducted to assess the difference between motion condition both within subjects (*group analysis*) and at the subject level (*individual analysis*). For both levels, we applied nonparametric tests, since we did not have any priors on the data distribution for inertia and MPC. For group analysis, statistical tests were conducted using 10,000 permutations on a nonparametric approximate (Monte Carlo) Friedman test for inertia, and if significant differences were inferred, approximate (Monte Carlo) Wilcoxon signed-rank tests were used for pairwise comparisons between conditions (with a decision criterion at p=0.05/3=0.017). For MPC, a Wilcoxon signed-rank test was carried out. All these tests were delivered using bootstraps based on 10,000 permutations conditional on subjects for every experiment (Necker, Cross, and Square) and metrics ($I_{\text{stimulus}}$, $I_{\text{fixation}}$, and MPC) using the packages *coin* (Hothorn, Hornik, Van DeWiel, & Zeileis, 2006) and *rstatix* (Kassambara, 2020). Effect size was computed from the $\chi^{2}$ statistics and using the transformation described by Tomczak and Tomczak (2014) to get a Kendall *W*, which varied between 0 and 1, with 1 the maximum effect size:

(3) $$W=\frac{\chi^{2}}{N(k-1)}.$$

with W Kendall's *W* value, $\chi^{2}$ the Friedman test statistic value, N the sample size, and k the number of measurements per subject. For each test, we report the $\chi^{2}$ Friedman test statistic, with the *p*-value (p) computed with the bootstrap, its effect size (Kendall *W*). For individual statistical analyses, we carried out an approximate Kruskal-Wallis test for inertia and an approximate Wilcoxon-Mann and Whitney test for MPC and pairwise comparisons using the same bootstrap package, with 10,000 permutations. To compare experiments’ data, Kruskal-Wallis tests were used over the three experiments’ RW and LJ data, respectively, and Wilcoxon-Mann and Whitney tests were used to infer differences between pairs of experiment data sets in each condition, with the same packages.

### Results

#### Micro-saccades

We described peak velocities, amplitudes, and rate of occurrences of micro-saccades detected during fixations (n=21,197, for Necker), using the algorithm from Engbert and Kliegl (2003). Distributions of micro-saccades’ peak velocities and amplitudes across conditions and experiments are shown in Figure 2A. Detected micro-saccades showed similar main sequences across motion conditions. Moreover, when we added the MPC value of the fixation in which the micro-saccade was detected (color scale), we observed (i) a higher prevalence of fixations with high similarity between gaze and predictable motion (LJ) than in the random walk (RW) condition, and (ii) no apparent (qualitative) correlation between MPC and micro-saccadic properties can be established. Micro-saccade rates are described in Figure 2B, with bootstrapped 95% confidence intervals.

When fixations with detected micro-saccades were kept, data preprocessing led to the removal of 12.32% of fixations for the Necker experiment based on fixation duration and outlier removal based on inertia with respect to screen. When fixations with detected micro-saccades were removed, data preprocessing led to the removal of 63.39% of fixations. Results presented next were computed on fixations not containing micro-saccades, as they describe the purest form of micro-pursuits. However, when including fixations containing micro-saccades, results led to the exact same conclusions.

#### Inertia and MPC

**Figure 3.**
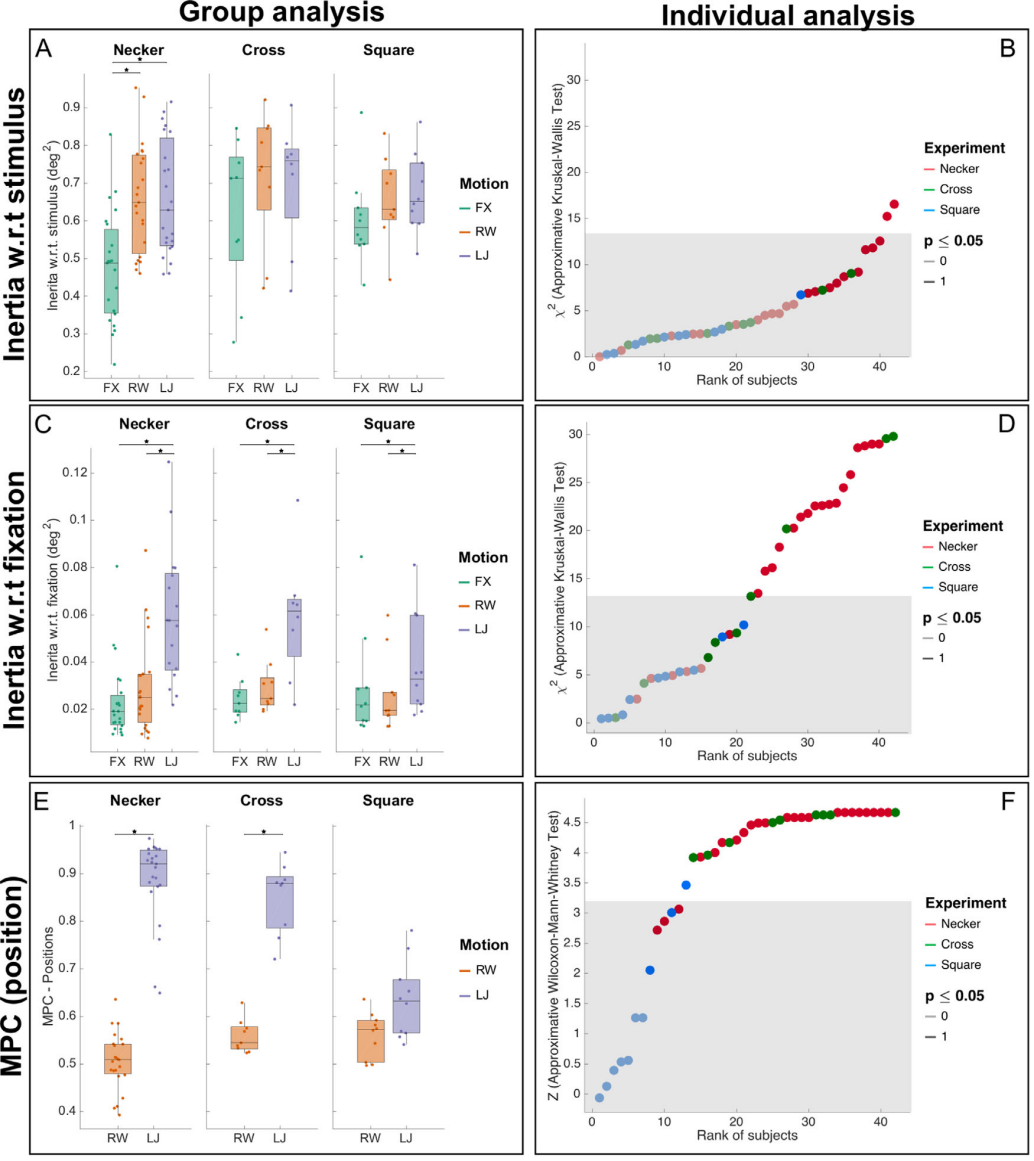
Micro-pursuit analysis. (A) A box plot of $I_{\text{stimulus}}$ over the three experiments (Necker, Cross, and Square) and three motion conditions (FX, RW, and LJ). Stars represent significant differences in pairwise comparisons using the Wilcoxon-Mann-Whitney test in a bootstrap. (B) The individual analysis results for $I_{\text{stimulus}}$ in all three experiments’ participants using an approximate Kruskal-Wallis test in a bootstrap. All participants have significant (p<0.05) results. For individual analysis, statistics (*z* score or $\chi^{2}$) that fall inside the 95% confidence interval were drawn with light color, whereas statistics values outside the 95% confidence interval were drawn in plain color. The gray area defines a conservative confidence interval corrected for multiple comparisons (Bonferroni), that is, 42 comparisons for the 42 tests computed on each subject. (C) A box plot of $I_{\text{fixation}}$ over all experiments and conditions. (D) The individual analysis results for $I_{\text{fixation}}$. The outcome of the statistical test per participant is given through different lightness values, with 1 (darker) meaning p≤0.05 and 0 (lighter) the opposite. (E) A box plot of MPC ($\rho^{*}$) over all experiments and the RW and LJ motion conditions. (F) The individual analysis results for $\rho^{*}$ in all participants using an approximate Wilcoxon-Mann-Whitney test.

We looked at the impact of the cube motion on eye movement and retinal image displacement. The former is made explicit through the inertia of gaze with respect to its average position within a fixation (see Figure 3B), whereas the latter is given by the inertia of the gaze with respect to the stimulus’ center of gravity (see Figure 3A). Descriptive statistics and statistical tests’ summary are given in Table 1.

**Table 1.** *Left*, Summary statistics of three measures for the Necker experiments in the FX, RW, and LJ motion conditions; inertia with respect to (w.r.t.) stimulus center of gravity ($I_{\text{stimulus}}$), inertia w.r.t. fixation center of gravity ($I_{\text{fixation}}$), and MPC ($\rho^{*}$). For each condition in the Necker experiment, median values over participants’ data are given with median absolute deviation following the ± sign. *Right*, Approximate Friedman test results ($\chi^{2};p$) and size effect (W) are given.

Necker (N=23)	FX	RW	LJ	$\chi^{2}$	p	W
${\tilde{I}}_{\text{stimulus}}$	0.488±0.189	0.649±0.190	0.629±0.159	23.565	<0.0001	0.512
${\tilde{I}}_{\text{fixation}}$	0.019±0.009	0.024±0.015	0.071±0.051	37.130	<0.0001	0.807
${\tilde{\rho }}^{*}$	n/a	0.509±0.048	0.921±0.047	23	<0.0001	1

Dispersion of eye movements around the fixation, computed with median inertia of the eye with respect to mean fixation position ($I_{\text{fixation}}$; see Figure 3C), differed with motion condition ($\chi^{2}=37.130;p<0.0001;W=0.807$). Paired comparisons of $I_{\text{fixation}}$ showed differences between FX, RW, and LJ ($Z_{FX-RW}=-2.4027,p=0.016;Z_{RW-LJ}=-4.1973,p<0.0001$; and $Z_{FX-LJ}=-4.1973,p<0.0001$). Thus, when computing retinal image displacement, we found that the median inertia differed across cube motion conditions (see Figure 3A). Indeed, we found a difference in inertia computed with respect to the center of gravity of the stimulus ($I_{\text{stimulus}}$) with motion condition ($\chi^{2}=23.565;p<0.0001;W=0.512$). Median inertia differed in the conditions where the stimulus was in motion ($Z_{FX-RW}=-3.9844,p<0.0001;Z_{FX-LJ}=-3.9539,p<0.0001$; and $Z_{RW-LJ}=0.09124,p=0.9445$).

When considering that stimulus inertia was equivalent for both motion conditions (${\bar{I}}_{\text{screen}}^{RW}=0.2995\pm 0.1988,{\bar{I}}_{\text{screen}}^{LJ}=0.2747\pm 0.1372$), the results suggest that both types of motion applied on the stimulus generated different effects on eye movements. Indeed, eye trajectories were more similar in the predictable LJ motion condition (${\tilde{\rho }}_{LJ}^{*}=0.921\pm 0.047$) than in the unpredictable RW motion condition (${\tilde{\rho }}_{RW}^{*}=0.509\pm 0.048$) with significant differences ($\chi^{2}=23;p<0.0001;W=1$ and $Z_{RW-LJ}=-4.1972;p<0.0001$). The data are reported in Figure 3E. We evaluated the effect of the cube motion for every subject and found similar results (Figure 3B, F) that will be described in more detail later.

#### Binocularity and velocity

**Figure 4.**
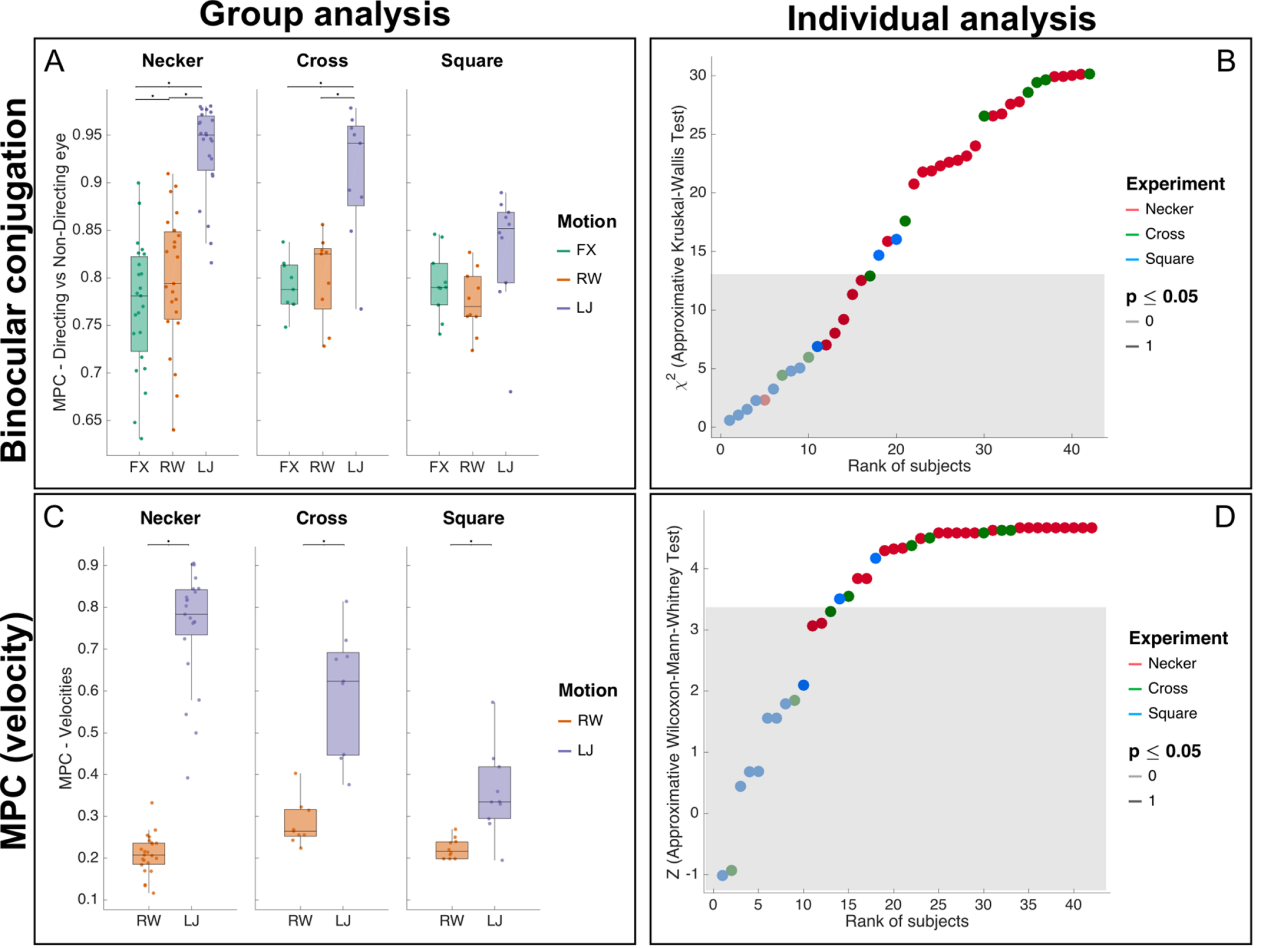
Micro-pursuit additional analyses. (A) A box plot of directing versus nondirecting eye similarity over the three experiments (Necker, Cross, and Square) and three motion conditions (FX, RW, and LJ). Stars represent significant differences in pairwise comparisons using the Wilcoxon-Mann-Whitney test in a bootstrap. (B) The individual analysis results for directing versus nondirecting eye similarity in all three experiments’ participants using an approximate Kruskal-Wallis test in a bootstrap. For individual analysis, statistics (z score or $\chi^{2}$) that fall inside the 95% confidence interval were drawn with light color, whereas statistics values outside the 95% confidence interval were drawn in plain color. The gray area defines a conservative confidence interval corrected for multiple comparisons (Bonferroni), that is, 42 comparisons for the 42 tests computed on each subject. (C) A box plot of MPC based on velocity vectors over all experiments and conditions. (D) The individual analysis results for MPC based on velocity vectors. The outcome of the statistical test per participant is given through different lightness values, with 1 (darker) meaning p≤0.05 and 0 (lighter) the opposite.

As binocularity is an important criterion that can discriminate between erratic noisy movement and conjugate and functional movement (Fang, Gill, Poletti, & Rucci, 2018), we also looked at the similarity of gaze between the directing and non-directing eye, to look at how conjugated the eyes were. We found overall differences across conditions ($\chi^{2}=37.130;p<0.0001;W=0.807$). Paired comparisons of eye versus eye similarity showed differences between FX, RW, and LJ ($Z_{FX-LJ}=-4.1973,p<0.0001;Z_{FX-RW}=-2.2202,p=0.023$; and $Z_{RW-LJ}=-4.1973,p<0.0001$). Results are reported in Figure 4A, along with analyses for each participant in Figure 4B.

To further investigate the pursuit description, we computed the MPC on the velocity signals, calculated on the position signals, down-sampled at 75 Hz, over six samples. In fact, as for the position analysis, LJ's predictable motion (${\tilde{\rho }}_{LJ}^{*}=0.798\pm 0.096$) led to higher velocity similarity between the eyes and the target than for RW's unpredictable motion (${\tilde{\rho }}_{RW}^{*}=0.246\pm 0.052$) with significant differences ($\chi^{2}=23;p<0.0001;W=1$ and $Z_{RW-LJ}=-4.1973;p<0.0001$). The data are reported in Figure 4C, along with analyses for each participant in Figure 4D.

### Intermediary discussion

When looking at our descriptive statistics (Table 1 and Figures 3A–C), participants’ median similarity based on MPC is centered on values of high correlation in the predictable motion condition (LJ) compared to the other motion condition (RW). This means that fixational eye movement gaze trajectories were, for most subjects, highly similar to that of the stimulus moving on screen. On the other hand, the unpredictable motion condition (RW) led to much lower similarity measurements, an observation that can be explained by the incapacity of the oculomotor system to predict the motion of the Necker cube as motion followed random walk dynamics.

Therefore, globally, participants’ gaze was influenced by the cube motion significantly more in LJ, where motion was predictable, than in RW, where motion was unpredictable, even though the oculomotor instructions were to fixate the cross in the middle of the screen for both. Moreover, the gaze in LJ showed similarity to the stimulus trajectories. All these measures were gathered on gaze data within fixation events, and the difference between LJ and RW conditions shows that oculomotor drift alone, as defined above, within fixational eye movements cannot account for this similarity. The oculomotor system would have to integrate visual information in order to quasi-systematically track the stimulus. We therefore refer to these detected fixational eye movements as micro-pursuits, in an effort to keep the analogy with the micro-saccades, while respecting the definition and metrics given above. Given the nondedicated and unpredicted observation of the oculomotor phenomenon in the Necker experiment, we carried out a second set of experiments to replicate the generation of micro-pursuit using a simpler stimulus and to verify that the phenomenon is not caused by the presence of a bistable stimulus—namely, the Necker cube.

## Replication experiments: Square and cross

The experimental protocol is similar to the previous one (Necker experiment) except that the Necker cube is replaced by a gray square and subjects have to report changes in luminance in either the fixation cross (Cross experiment) or the square (Square experiment). In the Cross experiment, we set the participants’ tasks and stimuli such that they had to follow a moving cross and detect changes of luminance on it. In the Square experiment, the setup aimed to investigate whether a complete reproduction of the Necker experiment, with a square instead of the Necker cube, would still lead to the observation of micro-pursuits.

### Method

Material and stimuli were identical to the previous experiment unless specified.

#### Apparatus

The stimulus was displayed on a 36-cm x 27.5-cm (19 inches) Dell M993s CRT screen of resolution 1,280 x 1,024 pixels and a 75-Hz refresh rate, located 57 cm from the participants, with white luminance at 70.89 cd.m^−2^, black at 0.09 cd.m^−2^, and mean gray at 15 cd.m^−2^. Eye tracking was done using an EyeLink 1000+ (SRT Research). Calibration was applied using a 5-point procedure between each block and if drift correction failed. Drift correction was applied between each trial. Participants had their head stabilized by sitting and resting their chin on a rest and their forehead against a bar.

#### Stimulus and motion conditions

As in Experiment 1, we replicated the three motion conditions (FX, RW, and LJ) using the same parameters with balanced mean inertia. Trials lasted 34 s (the mean time duration of *Experiment 1: Necker cube*) in which the same fixation cross was presented, and a moving object followed its trajectories depending on the condition (see Figure 1A).

#### Tasks and participants

The participants had to fixate a fixation cross surrounded by a square (2.5 deg x 2.5 deg), displayed in Figure 1A. They also had a perceptual task in which they had to report luminance changes using the same keys of the keyboard as in the Necker experiment. However, here the alternations were randomly selected among five levels of luminance (levels at 30%, 40%, 50%, 60%, and 70% of white) and duration of a level was selected using a log-normal probability law Log-N∼(μ=1,σ=1) seconds (see Figure 1C for a schematic representation of luminance over time). Two conditions were contrasted:

(1) *Implicit pursuit—moving Square luminance change detection*: fixate the fixation cross at the center of screen and report changes in luminance of the surrounding square moving with the three types of motions.
(2) *Explicit pursuit—moving Cross luminance change detection*: fixate the fixation cross and report changes in luminance of the fixation cross moving with the three types of motions.

The 19 participants (17 females and 2 males; age range = 18–30 years, μ=20.63±2.61 years), with normal or corrected-to-normal vision, were randomly oriented in one of the two experiments (Cross, n=9; Square, n=10) and provided their informed written consent before participating in the study, which was carried out in accordance with the Code of Ethics of the World Medical Association (Declaration of Helsinki) for experiments involving humans and approved by the ethics committee of University Grenoble Alpes. We estimated the number of participants to be included in the protocol based on a power analysis using g*power (Faul, Erdfelder, Buchner, & Lang, 2009) with α=0.05 and 1-β=0.95. We found that we needed a minimum sample size of nine participants (with 45 trials) to replicate the observations with a power of 0.95.

#### Data analysis

Data analyses were identical to the previous experiment.

### Results

The data were analyzed by applying the same signal-processing procedures and statistical methods as in the Necker experiment for inertia or MPC. When fixations with micro-saccades were kept, data preprocessing led to the removal of 8.79% and 9.23% of fixations for the Cross and Square experiments, respectively, based on fixation duration and outlier removal for inertia with respect to screen. Micro-saccade analysis (Figure 2) led to the extraction of main sequences with patterns showing no apparent qualitative differences between experiments (Necker, Cross, and Square) for amplitude and peak velocity, across motion conditions (FX, RW, and LJ).

When fixations with micro-saccades were removed as well, data preprocessing led to the removal of 65.43% and 72.73% of the data, in Cross and Square, respectively. Results presented in this section were computed on the fixations without micro-saccades, but when doing these analyses with fixations with micro-saccades, results led to the same conclusions.

#### Cross experiment: Explicit micro-pursuits

When participants had to explicitly follow the fixation cross, on which the motion and luminance signals were applied, similar patterns to the Necker experiment were found for inertia of gaze. Dispersion of eye movements around the fixation, computed with median inertia of the eye with respect to mean fixation position ($I_{\text{fixation}}$; see Figure 3C), differed with motion condition ($\chi^{2}=8.667;p=0.0096;W=0.481$). Moreover, paired comparisons revealed differences between FX, RW, and LJ ($Z_{FX-RW}=-2.403,p=0.016;Z_{RW-LJ}=-2.5471,p=0.0083$; and $Z_{FX-LJ}=-2.5471,p=0.0085$). Retinal image displacement differed with cube motion (see Figure 3A). We also found no significant difference in inertia computed with respect to the center of gravity of the stimulus ($I_{\text{stimulus}}$) with motion condition ($\chi^{2}=4.667;p=0.103;W=0.704$).

Given the fact that stimulus inertia was equivalent for both motion conditions, this suggests that motion of the stimulus generated different effects on eye movements. Indeed, eye trajectories were more similar in the predictable LJ motion condition (${\tilde{\rho }}_{LJ}^{*}=0.880\pm 0.050$) than in the unpredictable RW motion condition (${\tilde{\rho }}_{RW}^{*}=0.545\pm 0.032$) with significant differences ($\chi^{2}=9;p=0.0039;W=1$ and $Z_{RW-LJ}=-2.6656;p=0.0043$). The data are visualized in Figure 3E. We evaluated the effect of the cube motion for every subject and found similar results (Figure 3F).

#### Square experiment: Implicit micro-pursuits

Dispersion of eye movements around the fixation, computed with median inertia of the eye with respect to mean fixation position ($I_{\text{fixation}}$; see Figure 3C), differed with motion condition ($\chi^{2}=8.6;p=0.0109;W=0.43$). Moreover, paired comparisons revealed a difference between RW and LJ ($Z_{RW-LJ}=-2.3953;p=0.0126$) but not with FX ($Z_{FX-RW}=0.866;p=0.4321$ and $Z_{FX-LJ}=-1.8857;p=0.0609$). But retinal image displacement differed with cube motion (see Figure 3A). Indeed, we did not find a difference in inertia computed with respect to the center of gravity of the stimulus ($I_{\text{stimulus}}$) with motion condition ($\chi^{2}=2.4;p=0.3621;W=0.12$).

Given the fact that stimulus inertia was equivalent for both motion conditions, this suggests that motion of the stimulus did not generate different effects on eye movements. Unlike in the other experiments, eye trajectories were not more similar to stimulus trajectories in the predictable LJ motion condition (${\tilde{\rho }}_{LJ}^{*}=0.637\pm 0.097$) or in the unpredictable RW motion condition (${\tilde{\rho }}_{RW}^{*}=0.573\pm 0.044$) with no inferred statistical difference ($\chi^{2}=1.6;p=0.3384;W=0.16$). The data are visualized in Figure 3E. We evaluated the effect of the cube motion for every subject and found similar results (Figure 3F).

#### Individual analyses

We conducted the same analysis on every subject, and results are displayed for the three experiments and three motion conditions (Figure 3B, F). For every subject, we plotted the $\chi^{2}$ or z score statistics for the approximate Kruskal-Wallis and Wilcoxon-Mann-Whitney tests against their overall rank according to these statistics. For all subjects, we observed a main effect of inertia with reference to the stimulus ($I_{\text{stimulus}}$, with identical inertia between LJ and RW compared to FX). When looking at retinal displacement, we found the same pattern of result, that is, a main effect of motion, where inertia with reference to the fixation ($I_{\text{fixation}}$) is similar for FX and RW conditions but lower to LJ in the Necker and Cross experiments. For the Square experiment, results were mixed within subject, suggesting idiosyncratic behaviors. Finally, we observed more similar gaze pattern (high MPC) for the LJ condition both in the Necker and Cross experiments for every subject (except one out of nine in Cross) but mixed results for the Square experiment. Thus, individual analyses show that results observed at the group level are replicated at the subject level.

## Comparing Necker, Cross, and Square experiments

To summarize, descriptive statistics of detected micro-saccades in terms of main sequences (amplitude and peak velocity; see Figure 2A) and micro-saccade rates (Figure 2B) show that overall, micro-saccades are consistent across Necker, Cross, and Square, for all motion conditions. However, the Cross and Necker predictable (LJ) condition data seem to exhibit a different behavior from the other conditions and experiments when looking at gaze-target similarity (MPC). The micro-saccades’ fixation MPC displays many high correlation values, in contrast to the other conditions, and unlike the LJ condition in the Square experiment.

**Figure 5.**
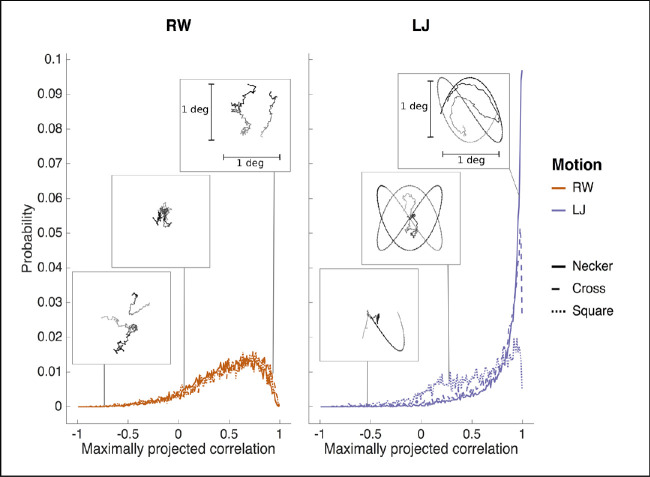
Focus on MPC results. Histogram of fixations by maximized correlation $\rho^{*}$ (MPC) scores in the Necker cube experiment. Illustrations of signals for values in some typical score intervals are presented to give a graphical intuition of the computed measure. We picked high similarity near a score of 1, no correlation near 0, and anticorrelation near -1. Dotted trajectories correspond to stimulus signals and continuous trajectories correspond to gaze signals. Temporal discourse is represented by lighter to darker samples.

Figure 5 provides a focus on MPC for fixations in all data sets, as well as for some selected signals that showcase some typical examples of gaze-stimulus pairs for different values of MPC. Since one cannot track the RW movements, the distribution of MPC under this condition serves as a baseline or null hypothesis control distribution. It can be seen that under RW, the empirically observed MPC distributions for all three experiments are confounded, indicating independence of MPC with respect to the experiment. Furthermore, it is also possible to observe a bias—the distribution is skewed toward the maximum value of 1—introduced by (i) the maximization of the correlation through the projection of the data into another coordinate system and (ii) the RW movement being low-pass filtered by the observer, and hence there exists a correlation at longer time scales. Indeed, the distribution under RW is not symmetric about 0 as would be the case for mere correlation between variables of multivariate independent Gaussian processes. On the other hand, under the LJ condition, the distribution skews even further to 1, resulting in a high probability for MPC values near 1, specifically in Necker and Cross. This is less so in Square.

When we removed fixations with detected micro-saccades and carried out inertia and MPC analyses, we found a difference for MPC in the LJ condition across experiments ($\chi^{2}=20.876;p<0.0001$). When looking at pairwise comparisons (subscripts N for Necker, C for Cross, and S for Square), no significant differences were found between Necker and Cross ($Z_{N-C}=-1.6136;p=0.106$), but Square differed from the other two ($Z_{S-C}=3.4293;p=0.0002$ and $Z_{N-S}=4.1915;p<0.0001$).

For RW interexperiment comparisons, we found an overall difference ($\chi^{2}=10.617;p=0.0036$). Paired comparisons showed a difference between Necker and the two other experiments ($Z_{N-C}=2.955;p=0.0020$ and $Z_{N-S}=-2.076;p=0.0350$) but none for Square versus Cross ($Z_{S-C}=1.061;p=0.3114$).

Finally, results for individual analyses show that most participants in the Square experiment had no significant differences between MPC in RW and LJ, while on the contrary, all 23 participants in the Necker and 8 out 9 participants in Cross did.

Overall, these results indicate that Cross did replicate the micro-pursuit phenomenon observed in the Necker experiment even with a smaller sample size, while Square did not.

Median inertia with respect to the stimulus’ center of gravity (${\bar{I}}_{\text{stimulus}}$) differed with motion conditions, suggesting that the nature of stimulus motion, manipulated in each condition (fixed, unpredictable, and predictable), affects global spatiotemporal dynamics of fixational eye movements. Median inertia with respect to the fixation's mean gaze position (${\bar{I}}_{\text{fixation}}$) showed the emerging pattern of a common oculomotor phenomenon occurring in Necker and Cross, where differences across conditions were measured. Again, this was not the case in Square (see Figure 3C). When looking at similarity between stimulus and gaze trajectories, integrated over fixation events using MPC, we found that the predictable motion condition (LJ) generated highly similar gaze trajectories in the Necker and Cross experiments, with large effect sizes. But we did not observe the same pattern for the Square experiment (see Figure 3E).

The contrast given by diverging results (Necker-LJ and Cross-LJ being different from Square-LJ) is interesting as it gives us a graduation of how likely the same predictable motion (LJ) can make observers generate micro-pursuit. It also suggests a coupling between the oculomotor and cognitive systems in the occurrence of micro-pursuits, which could be predicted and interpreted by a modeling framework we proposed when encountering the original observations. To go further, we propose a model, in Appendix C, that can describe all fixational eye movements in a single mechanism and can take into account the competition between multiple stimuli.

## Discussion

### Micro-pursuits

Given our definition of micro-pursuits (see Micro-pursuits section), which was based on an extrapolation of results available from the literature and our hypothesis of an oculomotor continuum, we have now gathered sufficient evidence to validate—at least partially—our proposed working definition. We believe that the following characteristics are elementary building blocks in defining micro-pursuits as a class of oculomotor movements or fixational eye movements:

#### 

##### Tracking or similarity with target

Probably the most prominent characteristic of micro-pursuits. When measuring similarity between the stimulus and gaze along the direction of maximum similarity using the MPC, we are able to categorize fixations as micro-pursuits, whether or not they contain micro-saccades. In addition, our proposed measure of similarity is invariant to scale, translation, and uncorrelated additive noise, compensating respectively for competition between fixation and tracking of a moving target as well as for instrumental or oculomotor drift and for acquisition noise. When the subject's gaze stays localized within the fixation (Square, all conditions), MPC indeed indicates that Square-LJ no longer has gaze following up on the target motion, contrary to Necker-LJ and Cross-LJ (see Figure 3).

##### Velocity and acceleration

Based on the literature review (Martins, Kowler, & Palmer, 1985; Skinner, Buonocore, & Hafed, 2018), all velocities of our stimuli were kept below 2 deg.s^−1^. At these velocities, we detected potential candidates of micro-pursuits, especially when the acceleration was moderate (LJ) (Figure 3) (MPC; Necker and Cross). When the acceleration was too high (RW^5^), micro-pursuits were no longer produced (Figure 3) (MPC; Necker, Cross, and Square). This advocates for the inclusion of both velocity and acceleration into the definition.

##### Binocularity

Binocular conjugacy is an essential ingredient if micro-pursuit is to be interpreted as an expression of a central control over the oculomotor system. Our results show that micro-pursuits in our experiments appear conjugated both at the group and at the individual level (see Figure 4).

In contrast to the above, the following elements of our working definition are no longer retained in our final proposition for a definition of micro-pursuits:

##### Amplitude

Given we focus solely on fixational eye movements, we found that a category of movements follows the below characteristics while staying under 1 deg in amplitude. However, if the oculomotor continuum holds, amplitude should no longer be a characteristic trait of (micro-)pursuit.

##### Duration

Although initially thought to be a defining characteristic of micro-pursuits, duration is a mere operational limitation. Indeed, the oculomotor system exhibits mechanical inertia and is thus intrinsically limited in its velocity and acceleration, resulting in trajectories with long autocorrelation times. Hence, for short observation periods, one has insufficient variability to accurately estimate *similarity*, independent of the method used.

In this work, we focused on a proof of micro-pursuits’ existence through the results obtained from the Necker experiment as well as through results from the replication experiments (Cross or Square).

Although the above results are obtained by retaining only fixations that do not contain any micro-saccades—as such being maximally conservative—our conclusions generalize when we include fixations with micro-saccades. As far as the micro-saccades are concerned, our data (presented in Figure 2) show main sequences that are invariant with respect to conditions and experiments. Furthermore, when looking at their marginal amplitude and peak velocity distributions, no clear differences can be observed across conditions and experiments. A similar observation can be made regarding their rate of occurrence. Also, micro-saccades within fixations that show pursuit behavior (high MPC values) show similar characteristics to those that are found in other fixations, since the MPC statistic do not correlate with either the peak velocity or amplitude of the micro-saccade under study. This provides evidence about the fact that slow fixational eye movements—tagged micro-pursuits in our work—can indeed be punctuated by micro-saccadic movements within a single fixation, and these do not interfere with the overall trend of the micro-pursuit movement. If the oculomotor continuum hypothesis indeed holds true, these micro-saccades could be associated with catch-up saccades. Unfortunately, due to our limited spatial resolution (video-based gaze tracker), we can not provide any further evidence for these.

Indeed, eye movement research is gradually considering an *oculomotor continuum*. For instance, it is becoming less and less credible to consider a hard separation between micro-saccades and saccades because of their common neural origins in oculomotor programming (Krauzlis, Goffart, & Hafed, 2017), their common properties, and mathematical models that can account for both (Sinn & Engbert, 2016). One may thus also consider that micro-pursuits share physical properties as well as neural correlates with large-amplitude smooth pursuits.

Alternative interpretations might classify fixations showing high inertia (w.r.t. fixation) as ocular drift. However, drift is considered independent from the stimulus and hence should not showcase high values of MPC as in the Cross-LJ and Necker-LJ conditions.

A limitation of this work is that it does not explicitly contrast experimental stimuli that are known to generate pursuit versus OFR. Indeed, as presented in the introduction, OFR are reflexive eye movements generated using sudden changes of a wide-field image (Quaia, Sheliga, FitzGibbon, & Optican, 2012) and should thus appear invariantly w.r.t. our experimental settings, but the lack of replication in the Square-LJ condition discredits this hypothesis.

A limitation of our similarity measure MPC resides mostly in its variance and thus the number of (temporally correlated) samples needed to accurately measure similarity. This is illustrated in Appendix B. While, on the one hand, physical properties (amplitude, peak velocity) can be used to discriminate micro-pursuits from micro-saccades, on the other hand, functional characterization will help provide discrimination between drift, slow motor control, and micro-pursuit. Indeed, the first two may be slow fixational eye movements but have no requirement for target tracking, like pursuit, whereas the latter does.

**Figure 6.**
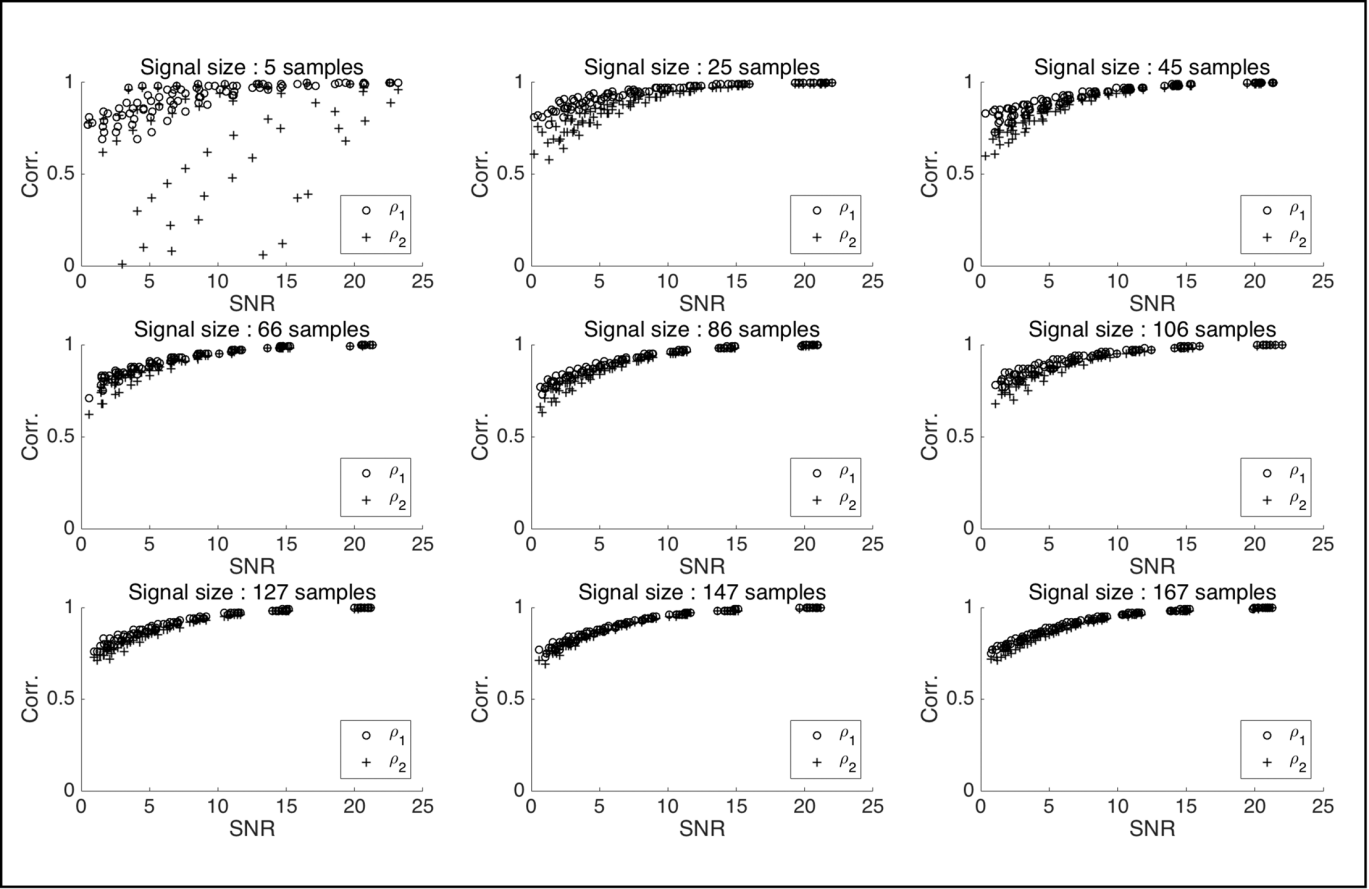
Behavior of MPC scores over signal-to-noise ratios (SNRs) in simulated similarity computations with a Lissajous base signal from LJ, with varying signal sample sizes.

Finally, micro-pursuits’ link to visual perception remains speculative, though interpreting our data suggests that the designation of the observed object, for perceptual report, and its associated motion (static, unpredictable, or predictable)—related to the distribution of cognitive capacities between perceptual and oculomotor tasks—may lead to a tentative explanation (Spering & Montagnini, 2011).

### Influence of oculomotor and perceptual tasks on target locking

In our two replication experiments, we have manipulated the task and target properties. In the Cross experiment, the task was to follow the moving object (cross) and to report its changes in illumination, while a static square was present in the background. In the Square experiment, the task was to fix a central fixation cross and report changes on the moving square object. Both have a similar relative movement of the cross with respect to the square object. In the first experiment, one can consider that participants had to focus on the cross. Whenever the latter was moving in a predictable, tractable fashion (LJ), the cross induced micro-pursuits. In the Square experiment, the competition between the perceptual and oculomotor tasks remained. Thus, one can consider the Square experiment to provide a competition between two attractors at the level of the oculomotor control, but given the reduced number of observed micro-pursuits (Figure 3E,F), one can interpret the competition between its attractors as unbalanced, where the fixation is more prominent than the follow-up on the moving target.

A first step toward a quantitative characterization of how a task may influence oculomotor dynamics is proposed in Appendix C. The proposed model is based on a competing attractor model inspired by gravitational field models. The model links the visual stimulation to perceptual objects modeled as gravitational attractors with dynamically varying masses, as such coping with attentional dynamics, whereas gaze position is modeled through a unit-mass particle subject to the gravitational field evolving in time. To account for perturbations and noise, velocity is subject to additive white Gaussian noise (Langevin dynamics). By manipulating the attractor's positions, masses, and the curvature of their *energy potential*, it is possible to generate (micro-)saccades, (micro-)pursuits, fixations, and drift. This mathematical model offers a quantitative method that may be interpreted in terms of spatial attentional loads, saliency, or intention, with respect to oculomotor programming and execution. It is an extension of some models already proposed in the field of fixational eye movements modeling based on energy potential (Engbert, Mergenthaler, Sinn, & Pikovsky, 2011; Herrmann, Metzler, & Engbert, 2017) as well as modeling work on bistable perception and processes (Moreno-Bote, Rinzel, & Rubin, 2007; Shpiro, Moreno-Bote, Rubin, & Rinzel, 2009; Moreno-Bote, Knill, & Pouget, 2011; Moreno-Bote & Drugowitsch, 2015), to incorporate the influence of, for example, ambiguous figures like the Necker cube.

### Future work

We proposed to use a set of metrics to detect micro-pursuit, but we need further experimental work to define the limits, the functional role, and the specificity of micro-pursuit with respect to other fixational eye movements.

First, discrimination between OFR and micro-pursuit can be assessed by contrasting stimuli with a variety of targets and backgrounds, for example, gratings (Gellman, Carl, & Miles, 1990). One may contrast pursuit capacity between tracked motion applied to a background texture and a target in the foreground.

Second, interaction between saccade and pursuit needs to be further studied. This can be done by varying speed and predictability of the target trajectory. When increasing velocity of the target, and under the oculomotor continuum hypothesis, pursuit movements will get interleaved with catch-up saccades that compensate for the accumulated retinal error (drift). Beyond a certain speed limit, a sequence of saccades and erratic movements—similar to those observed in our random walk condition or in the proposed simulation model—should be observed, indicating that micro-pursuits falter beyond an upper-bound velocity. However, we here attain the limits of our apparatus, and more precise and accurate eye-tracking methods are needed to determine whether specific catch-up micro-saccades do occur in micro-pursuit and in discriminating them from more generic micro-saccades.

Third, decreasing the predictability of the trajectories (increasing acceleration) will also lead to a transition from micro-pursuit over micro-pursuit, interleaved with micro-saccades, to erratic movement. One possibility is to tune noise and inertia (mass) for a stimulus position driven by Langevin dynamics as for the particle in our model.

Furthermore, manipulating the scale of the motion could provide insight into micro-pursuits’ link to large-amplitude smooth pursuit characteristics and may provide hints on its functional role.

Finally, the link between perception and oculomotor control of smooth pursuits has to be studied, for example, by varying the relative difficulty of the task (i.e., report changes) or the difficulty of the tracking. This might help in explaining the absence of positive results with respect to smooth pursuits within the Square experiment.

## Conclusion

In this work, micro-pursuits are proposed as a type of fixational eye movement occurring at small amplitude, within a fixation, as the gaze follows a target. We proposed two metrics: inertia and MPC to measure gaze displacement within a fixation and to quantify gaze-target trajectory similarity, respectively. We observed fixations in a predictable motion condition with higher gaze displacement and, more specifically, for both the Necker and Cross experiment data sets, fixations with high gaze-stimulus similarity values under predictable target trajectories for position and velocity analyses. Binocular conjugation of the reported observations also provided evidence supporting the existence of micro-pursuit fixational eye movements. Micro-pursuit here is presented as a class of fixation, but further research is needed to identify the physical properties and distinguish them from other fixational eye movements. Moreover, this article calls for further investigation on the functional role of micro-pursuits and how the oculomotor and perceptual systems interact during such movements.

## Figures and Tables

**Figure 7.**
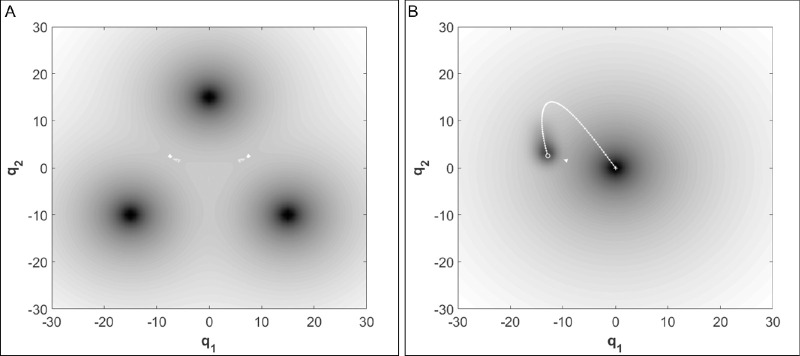
Examples of energy landscape surface plots setup using the gravitational fixational eye movements (GraFEM) model for the following: (A) shows three attractors (n=3) with all attractors i having no motion and the following parameters: β=2;δ=1;τ=5;λ=0.9, and (B) shows two attractors (n=2) with all attractors i having the following parameters: $\beta_{1}=2;\;\beta_{2}=4;\;\delta_{1}=\delta_{2}=1;\;\tau =15;\;\lambda =0.9$, and attractor motion computed with the following arbitrary sinusoidal motion: $a_{1}\left(t\right)=\left[0,0\right];\;a_{2}\left(t\right)=a_{2}\left(t=0\right)+\left[-5\sin\left(2t\right),5\sin\left(3t\right)\right]$ on the 75th iterations. The motion of $a_{2}$ is shown in white. Though the model has many parameters, those manipulated in this work's results are exclusively the depth δ (or mass) of the attractors and the slope β by affecting the concavity of the attractors’ field.

**Figure 8.**
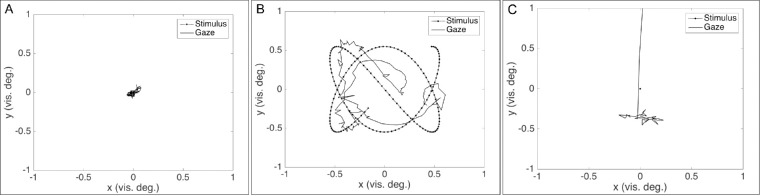
Simulation examples generated with the GraFEM model. Simulations of fixations of 3.5 s with Euler-Muruyama time steps of Δt=13 ms, with variable fixation dynamics generated by manipulating $\delta_{\text{stim}}$ and $\beta_{\text{stim}}$ parameters. Constant parameters of the model were number of attractors n=2, with one for the fixation cross $a_{\text{cross}}=\left(0,0\right)$ and another for the motion of the stimulus following Lissajous trajectories with the same parameters as in the three experiments (Necker, Square, Cross); $a_{\text{stim}}\left(t\right)=\left(\sin(2t),\sin(3t)\right)$. The relaxation rate parameter λ=0.9, memory temporal limit parameter K=5, and noise ξ∼U[-0.5;0.5] were used. (A) A simulated fixation with stable fixation dynamics with $\delta_{\text{cross}}=100;\;\delta_{\text{stim}}=100;\;\beta_{\text{cross}}=1;\;\beta_{\text{stim}}=1$. (B) A simulated fixation with micro-pursuit dynamics with $\delta_{\text{cross}}=100;\;\delta_{\text{stim}}=25;\;\beta_{\text{cross}}=1;\;\beta_{\text{stim}}=1$. (C) A simulated fixation with micro-saccade dynamics with $\delta_{\text{cross}}=100;\;\delta_{\text{stim}}=25;\;\beta_{\text{cross}}=1;\;\beta_{\text{stim}}=12$ and detected using the EK algorithm for micro-saccade detection.

**Figure 9.**
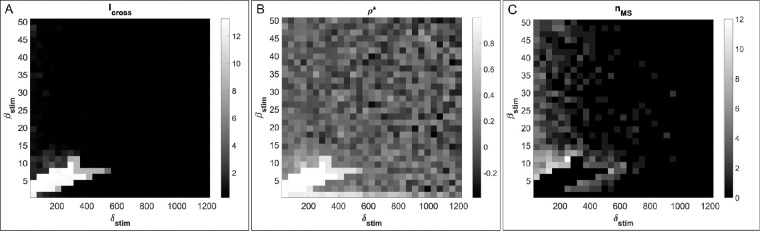
Simulations and analyses of the GraFEM model. Simulations of fixations of 1.5 s with Euler-Muruyama time steps of Δt=1 ms, with variable fixation dynamics generated through the variation of $\delta_{\text{stim}}\in \left[0;1200\right]$ and $\beta_{\text{stim}}\in \left[0;50\right]$ parameters. Constant parameters of the model were number of attractors n=2, with one for the fixation cross ($a_{\text{cross}}=\left[0,0\right]$) and another for the motion of the stimulus following Lissajous trajectories with the same parameters as in the Necker cube experiment: $a_{\text{stim}}\left(t\right)=\left(\sin(2t),\sin(3t)\right)$. The relaxation rate parameter λ=0.9, memory temporal limit parameter K=5, and noise ξ∼U[-0.5;0.5] were used. (A) The behavior of inertia over the parameter space of the GraFEM model. (B) The behavior of the similarity between stimulus and simulated fixation motion using the MPC $\rho_{1}$. (C) The number of micro-saccades detected by the EK micro-saccade detection algorithm.

**Figure 10.**
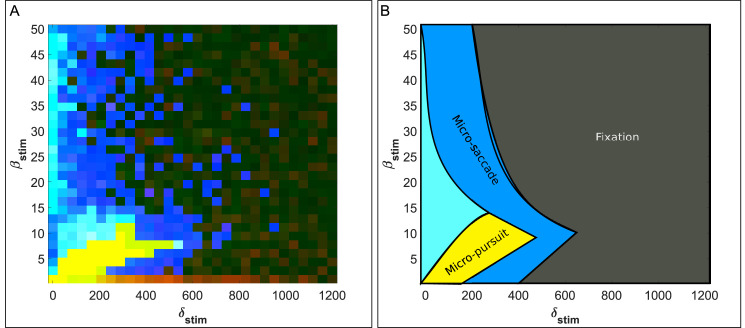
GraFEM oculomotor interpretation. (A) A merger of measures applied on GraFEM synthetic data. Simulated fixations’ inertia, MPC, and detected micro-saccade results are assigned to the red, green, and blue components of this RGB matrix, respectively. The yellow space provides β and δ values that generate micro-pursuit, the blue pixels show a micro-saccade generation transition space, the dark area shows stable fixation parameters, and the red pixels could be interpreted as slow control. (B) A schematic interpretation of oculomotor dynamics generated in the parameter space of the GraFEM model with manipulation of the $\delta_{\text{stim}}$ and $\beta_{\text{stim}}$ parameters of the stimulus attractor, while keeping all other parameters constant. Micro-pursuit can be generate in a restrained subspace (yellow) while micro-saccades are detected in the surrounding space (dark blue). When $\delta_{\text{stim}}$ values are low, a transition area (light blue) exists where micro-saccades and similarity are high, but inertia is not. Finally, stable fixation subspace occupies the rest (gray).

## Footnotes

1 For the use of the notations in this article, the reader is referred to Appendix A.

2 Here, we present the velocities rather than frequencies to provide comparable measures across reviewed articles. However, in most cases, the target signal corresponds to a sinusoidal movement; thus, velocity is not constant over a period.

3 The surface area of the ellipse such that the data belong to this area with a probability of 68% when a two-dimensional Gaussian fits the data; roughly speaking, up to a factor, it is the determinant of the empirical covariance matrix.

4 Here, data points refer to a statistic of a fixation period, for a given experiment, subject, and condition. We also have an outlier subject (71 years old) who is not removed.

5 Acceleration is due to rapid changes in the direction, as in the magnitude of the velocity vector, due to additive white Gaussian perturbations of the latter.

## Appendix A: Notations used

In this work, we use q and $\dot{q}$ for the two-dimensional position (in deg) and velocity vectors (in deg$\cdot {\text{s}}^{-1}$). Subscripts R, G, and S will respectively refer to the retinal image, the gaze, and the stimulus. Overlined notation will refer to the mean over a set of trials and a tilde to the median over a set of trials, for all metrics. Mean values will be reported with their standard deviation and median values with median absolute deviation (MAD), for instance, $\bar{\rho }=2.93\pm 0.01$ or $\tilde{\rho }=3.01\pm 0.02$.

## Appendix B: Metrics

We use $\cdot^{⊤}$ for the transpose operator, and trace(·) will denote the trace operator (sum over the diagonal elements of a matrix). The identity matrix in dimension 2 will be denoted by ${\mathrm{Id}}_{2}$.

### Variance-covariance and inertia

Let $q_{G}\left(t\right)={\left[x_{G}\left(t\right),\;y_{G}\left(t\right)\right]}^{⊤}$, $q_{S}\left(t\right)={\left(x_{S}\left(t\right),\;y_{S}\left(t\right)\right)}^{⊤}$, and $q_{R}={\left[x_{R}\left(t\right),\;y_{R}\left(t\right)\right]}^{⊤}$ be the screen Cartesian coordinates (column vectors) at time instant t of the gaze, the stimulus, and the retinal image, respectively. Now, having samples at n discrete times ${\left\{t_{i}\right\}}_{i=1}^{n}$, we estimate the center of gravity of a gaze trajectory ${\left\{q_{G}\left(t_{i}\right)\right\}}_{i=1}^{n}$ by its empirical mean $m_{G}=n^{-1}\sum_{i=1}^{n}q_{G}\left(t_{i}\right)$. This estimate approaches the true center of gravity if we sample sufficiently regularly and beyond twice the Nyquist frequency, conditions that are met when working with the Eyelink 1000(+), sampling at about 1,000 Hz for each eye.

A second-order statistic of interest is the empirical variance-covariance matrix, which gives the inertia of the gaze trajectory defined as

$$\Sigma_{G}=n^{-1}\sum_{i=1}^{n}q_{G}\left(t_{i}\right)q_{G}^{⊤}\left(t_{i}\right)-m_{G}m_{G}^{⊤}$$

and analogously for the stimulus and retinal image empirical variance-covariance matrix. The inertia about its center of gravity is then given by $I_{m_{G}}=n^{-1}\sum_{i=1}^{n}{∥q_{G}\left(t_{i}\right)-m_{G}∥}^{2}=\mathrm{trace}\left(\Sigma_{G}\right)$.

The inertia $I_{r}$ of the gaze trajectory $q_{G}$ with respect to any fixed point r having screen coordinates $\left(x_{r},\;y_{r}\right)$ is

$$I_{r}=\mathrm{trace}\left(\Sigma_{G}\right)+{\left(m_{G}-r\right)}^{⊤}\left(m_{G}-r\right).$$

### Maximally projected correlations

Taking now the simultaneously recorded gaze ${\left\{q_{G}\left(t_{i}\right)\right\}}_{i=1}^{n}$ and stimulus ${\left\{q_{S}\left(t_{i}\right)\right\}}_{i=1}^{n}$ signals, and their respective empirical variance-covariance matrices *G* and *S*, we denote the intercovariance matrix by

$$\;\;\;\;\;\;\;\;\;\;\Sigma_{GS}≐n^{-1}\sum_{i=1}^{n}\left(q_{G}\left(t_{i}\right)-m_{G}\right){\left(q_{S}\left(t_{i}\right)-m_{S}\right)}^{⊤}=\Sigma_{SG}^{⊤}.$$

This matrix is particularly useful when considering the inertia of gaze with respect to the time-changing coordinates of the stimulus. Indeed, after some manipulations, we obtain

$$\begin{array}{ccc} \;\;\;\;\;\;\;\;\;\;\;\;\;\;\;\;\;I_{GS} & \;= & n^{-1}\sum_{i=1}^{n}{∥q_{G}\left(t_{i}\right)-q_{S}\left(t_{i}\right)∥}^{2}\\ \;\;\;\;\;\;\;\;\;\;\;\;\;I_{GS} & \;= & \mathrm{trace}\left(\Sigma_{G}+\Sigma_{S}-\Sigma_{GS}-\Sigma_{SG}\right)+{∥m_{G}-m_{S}∥}^{2}. \end{array}$$

Unfortunately, the inertia does not account for differences in scale or for coordinate translation, two characteristics that are typical aspects for pursuits and for which we require an invariance.^6^

### Noise robustness and signal size dependency

Figure 6 shows results of simulations operated on a Lissajous signal degraded by noise on the position of the stimulus at different signal-to-noise ratios (SNRs) and for different signal sizes. For signals with more than 167 samples, the behavior of MPC scores over SNR remains stable and shows quasi-unchanged dynamics.

## Appendix C: Model

Models come in a variety of forms, depending on the mathematical framework used to formalize and compute their mechanics. Two main families can be differentiated: descriptive statistical and generative mechanistic models. Here, we focus on the latter. The motivation is the following: Generative models can produce simulated and synthetic results that can be compared to observed empirical data. The model can then be studied and decomposed such that each internal force can be characterized, and their functional role in creating the analogous behavior can be investigated. Altogether, models remain key to understanding a phenomenon and making predictions for empirical and experimental work. We focused here on fixational eye movements in an attempt to explain and understand the data observed and reported in the article. Generative eye movement models use different approaches, including, for instance, probabilistic models (Tatler, Hayhoe, Land, & Ballard, 2011; Gide & Karam, 2017), accumulation process models (Orquin & Loose, 2013), or energy potential models (Engbert, Mergenthaler, Sinn, & Pikovsky, 2011). Here we focus on the latter approach.

Recently, Engbert and colleagues (Engbert, Mergenthaler, Sinn, & Pikovsky, 2011) proposed a generative model that could reproduce the statistical properties of fixational eye movements, stationary displacement, namely, the short-term persistence and long-term antipersistence of drift and tremors. They used a self-avoiding walk (Freund & Grassberger, 1992) in a discretized quadratic energy potential: At each iteration, the gaze, represented by a particle in the energy potential landscape, can go left, right, up, or down. The walker will choose the slot with the lowest energy. Once a step is made, the slot of the previous iteration is set to a high-energy value, and the entire energy landscape follows a linear relaxation law. Hence, fixational eye movements, bottom-up dynamics can be reproduced. Furthermore, the model also proposed to integrate micro-saccade generation by a threshold rule: When the particle is surrounded by options with energy higher than the threshold, it jumps to the global minimum of the energy landscape. Here, the authors provide an accumulation process linked to a global integration of the oculomotor field.

The integrated fixational eye movements model described above is a key foundation to bridge the oculomotor modeling communities and accounts for multiple fixational eye movement phenomena (e.g., drift displacement, micro-saccade, spatial orientation biases). However, it did not possess a mechanism to account for micro-pursuit, as these are hardly studied and reported. The observation of micro-pursuits presented in the article implies that the dynamics of the gaze within a fixation can be affected and attracted by motion of a perceptual object in or nearby the foveal field. Therefore, we propose a modeling approach, gravitational fixational eye movements (GraFEM), inspired by gravitational energy field theory to model motion of eye movements and derived from the work on integrating fixational eye movements in energy potential models (Engbert, Mergenthaler, Sinn, & Pikovsky, 2011).

### Gravitational potential energy field modeling

Integrated and generative fixational eye movement models make use of energy potentials to generate self-avoiding walks, constrain the walks, and replicate oculomotor biases (Engbert, Mergenthaler, Sinn, & Pikovsky, 2011). In fact, the latter is used to constrain the pseudo-random walk's spatial horizon. Furthermore, it can be considered an attractor of the energy landscape. Thus, the use of the particle in an energy potential framework can be adjusted to provide biases of the stimulus on the fixational eye movement generation. Combining attractors in the energy fields, which increase the probabilities of having the gaze at some spatial coordinates, and adding stochasticity to the movement of the particle can provide a simple mechanism for fixational eye movement generation.

The attractors’ properties can be manipulated over time to affect the energy field and thus dynamics of the fixational eye movements generated. The energy field that is mapped to the visual field can be populated by an arbitrary number of n attractors of varying strength (see Figure 7A). Inspired by the formalism of gravitational fields, one can generate fields with the following equations. Let $\Phi_{i}$ represent the field generated by the *i*th attractor given by

(4) $$\Phi_{i}\left(q,t\right)=-\frac{1}{{∥q\left(t\right)-a_{i}\left(t\right)∥}^{2\beta_{i}\left(t\right)}+\delta_{i}\left(t\right)}$$

with q and $a_{i}$ corresponding to the spatial x-y coordinates (at time t) of the observer's gaze position and the *i*th attractor, respectively. The potential landscape can be fine-tuned according to assumptions on attractive attributes of the stimulus and the tasks. First, it is necessary to set how many attractors are present and give them spatial coordinates in the plane over time. Second, it is possible to handle the mass of those attractors and their subsequent force of attraction and distortion of the field by tuning two parameters: δ for the depth of the well and β for the concavity of its slope. Summation and normalization of the field allow for the fusion of the multiple attractors.

(5) $$\Phi \left(q,t\right)=\sum_{i=1}^{n}\Phi_{i}\left(q,t\right).$$

A logarithmic attenuation is added to allow the possibilities of exploring high-energy areas of the visual/foveal field, giving the energy E:

(6) E=-ln(-Φ).

Memory of attractor motion (Figure 7B) is modeled by adding a moving average (MA) process (Hannan, 2009) on the field at a given time t:

(7) $$\;\;\;\;\;\;\;\;\;\;\;\;\;E_{FEM}\left(q,t\right)=E\left(q,t\right)+\sum_{k=1}^{K}\frac{\lambda }{k+1}E(q,t-k\Delta t),$$

where K is the temporal parameter limiting how far in time will the fields be summed over and with λ the relaxation rate parameter, and Δt is the temporal step size. It is also possible to set the impact of memory and anticipation through parameters that define the iteration window over which the field is deformed using traces of the attractor in the past of a given current iteration and the rate λ at which the deformation affects for a given lag.

A particle of position (q) with negligible mass (or with very high friction) is dropped in the field and is disturbed by noisy force, in order to generate and simulate gaze dynamics. Therefore, given the fundamental relation for dynamics, where the accelerating second-order component is neglected, the gaze particle's motion is derived by the Langevin equation (Langevin, 1908), in which $m\ddot{q}$ is equal to the sum of forces applied to the particle, and can be rewritten as follows:

(8) $$m\ddot{q}=-\gamma \dot{q}-\nabla E_{FEM}\left(q,t\right)+\xi \left(t\right)$$

with m the negligible mass, γ the friction, and ξ an external force, here an oculomotor noise (η) applied to the gaze, such that $\eta \left(t\right)=\frac{\xi (t)}{\gamma }$. With the assumption of low mass and after normalization,^7^ such that $E_{FEM}=\frac{E_{FEM}}{\gamma }$, the dynamics can be expressed as

(9) $$\dot{q}=-\nabla E_{FEM}\left(q,t\right)+\eta (t).$$

The evolution of the gaze particle's dynamics can be computed by making the problem a discrete one using the Euler-Maruyama method (Kloeden & Platen, 2013), for instance.

### Model simulations: What are the parameters corresponding to ocular events and interpretation?

Fixations of 1.5 s, with a discretization Euler-Maruyama step Δt=1 ms equal to the time step, were simulated using the GraFEM model with two attractors,: $a_{\text{cross}}$ corresponding to the attractor of a fixation cross at the center and $a_{\text{stim}}$, the attractor representing the stimulus, with a Lissajous motion: $a_{\text{stim}}=\left(\sin\left(2t\right),\sin\left(3t\right)\right)$. Only the slope and depth parameters were manipulated: $\beta_{\text{stim}}\in \left[0;50\right]$ and $\delta_{\text{stim}}\in \left[0;1200\right]$. All other parameters were kept constant with the other attractor position at $a_{\text{cross}}=\left(0,0\right)$ with $\beta_{\text{cross}}=1$ and $\delta_{\text{cross}}=100$, the relaxation rate parameter λ=0.9, the memory temporal limit K=5, and noise ξ∼U[-0.5;0.5]. These simulated fixations were then analyzed using the measures presented in this article, namely, inertia, MPC, and micro-saccade detection using the Engbert-Kliegl (EK) algorithm based on relative velocity thresholds (Engbert & Kliegl, 2003). Figure 9A shows that higher inertia follows a diagonal region along the $\left\{\beta_{\text{stim}},\delta_{\text{stim}}\right\}$ space. When looking at Figure 9B, one can see that the same area in the parameter space has systematically high MPC. Finally, the EK algorithm was applied (without the binocularity criterion) to measure detected micro-saccades and summed over the time of a fixation. The results (Figure 9C) show that micro-saccades are detected when concavity is high due to a larger $\beta_{\text{stim}}$ parameter.

### Discussion and perspectives: Attractor, oculomotor, and perceptual multistability

The simulation results presented above show the following three points. First, fixations’ dynamics can be modeled, including a variety of fixational eye movements such as drift, tremors, micro-saccades, and micro-pursuits. Second, attractor dynamics can be intuitively manipulated by two parameters that control their slope and depth, hence imposing, by gravity, faster or slower dynamics on the gaze particle. Third, generalization to more complex stimuli or tasks can be maneuvered by such a model as attractors can be multiplied, if necessary. However, this work remains preliminary and calls for further investigation. Such perspectives are discussed in the following paragraphs.

#### Model interpretation for eye movements

The GraFEM model proposed in this article is capable of generating micro-saccade, drift, and tremor fixational eye movements (see Figure 8) as classified in the literature (Martinez-Conde, Macknik, & Hubel, 2004), as well as the micro-pursuits presented and detected in the article, as reported in Figure 9 and Figure 10. By using classified data (observations), the parameters of the model that allow the generation of these fixational eye movements could be inferred, and insights on the mechanics of micro-saccades, micro-pursuit, drift, and tremor generation and their interaction can be studied. The diagrams in Figure 10 already give a useful and overall understanding of the model, with respect to the manipulated parameters, but the work on parameter inference should be addressed in the near future in more details.

Given the observed data and the proposed model to account for it, questions and perspectives can be redefined with a novel angle for interpretation of fixational eye movements. Inversion and a full analysis of a model, like GraFEM, with multiple free parameters is a complex task out of the scope of this thesis but should be tackled and reported in the near future.

The model presented here gives a mathematical framework in which eye movement phenomena can be generated and interpreted. Attractors are interesting as tools to explain and interpret cognitive and physiological behaviors as they allow an intuitive understanding of the evolution of dynamical systems (Watanabe, Masuda, Megumi, Kanai, & Rees, 2014; Kelso, 2012). Furthermore, complex learning systems—that is, neural networks—are known to develop such properties as the parameters of their processes tend to learn the statistics of the environment by creating attractors in the parameter space (Moreno-Bote, Rinzel, & Rubin, 2007; Shpiro, Moreno-Bote, Rubin, & Rinzel, 2009; Moreno-Bote, Knill, & Pouget, 2011; Moreno-Bote & Drugowitsch, 2015).

With this modeling framework, the fixational eye movements classification of the literature can be described and interpreted in terms of attractor spatiotemporal dynamics (Figures 9 and 10).

A stable fixation (Figure 8A) in the GraFEM model corresponds to a stabilization of an attractor with the energy landscape having little change. The gaze particle is stuck, and only the noise affecting its position may lead to small random movements of the eyes, as in other generative fixational eye movement models (Engbert, Mergenthaler, Sinn, & Pikovsky, 2011; Herrmann, Metzler, & Engbert, 2017). In these models, constraints to the energy field of the fixation are used in an analogous fashion to reflect the higher probabilities of having fixational eye movements in horizontal and vertical directions. A fixation attractor can thus be predicted by the task or the stimulus controlled by the experiments, and its parameters can be inferred by a priori information and data. Hence, the model gives predictive capabilities that can be tested and requires assimilation of data to constrain its range of possibilities.

Micro-saccades (Figure 8C) correspond to sudden changes in the energy depth of attractors, with a new one emerging or deepening while the attractor of fixation has suddenly disappeared. They are likely to emerge as the gaze particle rushes down a gradient to the center of an attractor, giving it sufficient velocity. The depth and slope of the attractor can be manipulated (following the dynamics described in Figure 10), thus making it possible to infer, based on observed velocities and amplitudes, the saliency of that attractor. The GraFEM model does not use an explicit and separated mechanism for micro-saccade generation—as the model presented in Engbert, Mergenthaler, Sinn, and Pikovsky (2011)—though it is not incompatible.

Drifts correspond to a stability of the gaze particle with respect to the attractor by which it is transported. However, the attractor might itself slowly drift away in the visual space (independently from the target motion), or alternatively, the shape of the well might get larger (by manipulating the parameter β), allowing for the noisy gaze particle to explore further. These are two hypotheses that could be tested, in future work, by inferring the model parameters given sufficient data. These fixational eye movements are known to help reduce visual redundancy and extract features in complex visual stimuli (Kuang, Poletti, Victor, & Rucci, 2012) but are mostly considered consequences of the eye muscles and their neural control properties. Therefore, they have mostly been considered independent processes from the visual stimulus presented.

The micro-pursuits detected and described in the article could be interpreted as a form of stimulus-related drift, as its signal dynamics place it in similar ranges, and is captured by the proposed metric, namely, MPC. Consequently, this argues in favor of our proposition that drifts are composed of two categories stimulus independent and dependent and micro-pursuits logically fall within visually dependent ocular drifts. This dependency can be interpreted as the interference of bottom-up salient elements interrupting the top-down task of fixation. Micro-pursuits (Figure 8B) are therefore close to drifts in the energy landscape dynamics.

#### Model mechanics

The model sets the gaze as a particle in an evolving gravitational energy potential field. When the system has no dynamics added to the potential landscapes, the particle will fall into its nearest local minimum. In this implementation, at each iteration—here a discrete time step using Equation 9—the first derivative is computed to update the position of the particle in the plane, corresponding to the screen. Noise is then added to the deterministic dynamics and can drive fixational oculomotor decision-making with respect to attractors if its amplitude is sufficiently large (Shpiro, Moreno-Bote, Rubin, & Rinzel, 2009; Moreno-Bote, Rinzel, & Rubin, 2007). This mechanism is similar to bistable energy potential models, though it extends on the dimensions of the system. In a set of simulations reported in Figure 9, we show that through two continuous parameters applied to a target attractor, it is possible to generate and interpret oculomotor dynamics observed in fixational eye movements. However, here, there is no prior requiring the existence of different systems for each class of movements observed (Barnes, 2011). Fixational eye movement dynamics can be reproduced through a unique mechanism, as shown by the simulated examples in Figure 8.

Top-down intention processes can be tested and simulated, given the context of a task, by applying changes in the model's β and δ parameters. Figure 10 can be used as a road map of the oculomotor dynamics and regimes expected, depending on parameter values. Moreover, bottom-up saliency or attentional effects can also be taken into account. This can be done with simpler assumptions, such as the ones presented here for the task used in the article, but can be more complex if using natural scene tasks, for instance. An interesting and practical perspective in this context lies in investigating how salience models, which derive probability distribution based on the statistics of images or videos, can be integrated such that only attractors are fed into a GraFEM oculomotor execution system.

#### How would this be implemented in the brain?

Anatomically, oculomotor programming has been shown to be highly correlated and linked to a network of areas involving neural activity in the superior colliculus, the frontal eye field, and the lateral intraparietal cortex (Hafed, Goffart, & Krauzlis, 2009; Krauzlis, 2004, 2005; Krauzlis, Goffart, & Hafed, 2017; Astrand, Ibos, Duhamel, & Hamed, 2015; Peel, Hafed, Dash, Lomber, & Corneil, 2016; Taouali, Goffart, Alexandre, & Rougier, 2015). There are interindividual differences in anatomy and behavior for fixational eye movements measuring and observed dynamics. For instance, it has been shown that not only oculomotor behavior between trained and untrained participants varies a lot, but drift also accounts for more fixation correction motion than micro-saccades (Cherici, Kuang, Poletti, & Rucci, 2012). The observations of micro-pursuits presented in the article suggest that the dynamics of the gaze within a fixation can be affected and attracted by motion of an object in or nearby the foveal field.

However, rather than having an attractor with a pseudo-random displacement, its motion follows a deterministic and predictable trajectory, which can be computed and estimated by the oculomotor system. Moreover, that attractor is, given our observations so far, only related to a target motion. This could, for instance, be implemented in the brain by means of an efference copy (Astrand, Ibos, Duhamel, & Hamed, 2015), though this idea remains speculative, and further modeling and neurophysiological research are needed. The low energy attributed to a decoded and perceived object moving across space encourages the oculomotor system to track it as it tries to minimize the energy of the gaze particle. Finally, tremors are generated and explained by the noise given to the particle over all fixational eye movement events.

This model complements the eye movement field of research with the possibility to program intentions, salience, and their effects on the gaze dynamics by simply using attractors and setting out their dynamics in terms of motion on the visual field, depth, and memory. For instance, the model can predict the different dynamics reported based on the eccentricity of an attractor corresponding to an afterimage, as observed in Heywood and Churcher (1972). Thus, one can use the model to generate statistical predictions of eye movement dynamics. Given an understanding of the visual attention or saliency effects of their stimulus and taking into account all the associated intentions to the tasks that participants are required to operate during a trial, it is possible to use this modeling to generate quantitative predictions of the oculomotor dynamics. Moreover, the generative properties make it possible to work on simulated data and extract dynamics' statistics in terms of eye movements, and this is possible using the traditional algorithms for eye movements classification. Inversely, obtaining the parameters of the model that replicate the dynamics of observations could help understand better the internal processes that drive eye movements.

### Perspectives: Toward oculomotor multistability

A key aspect of this family of models is that it showcases *multistability* regarding their attractors. This phenomenon can emerge in many complex biological systems and is present in many cognitive processes (Schwartz, Grimault, Hup, Moore, & Pressnitzer, 2012). It is linked to coordination dynamics between subsystems that have varying levels of coupling, leading to monostable, multistable, or metastable dynamics (Kelso, 2012). The consequent interpretation is that the oculomotor system could have multistable dynamics with respect to visual attractors. In this case, the oculomotor dynamics are likely driven by noisy signals (Braun & Mattia, 2010) representing other interfering systems, such as perception, attention, intention, and other cognitive systems. This framework connects to the growing body of studies linking perceptual decisions and multistable system dynamics. It also creates a link for motor systems to studies of noise as a component that helps a perceptual system operate through stochastic resonance^8^ (Gammaitoni, Hänggi, Jung, & Marchesoni, 1998; Patel & Kosko, 2005; Kim, Grabowecky, & Suzuki, 2006).
